# Secrecy Analysis of a Mu-MIMO LIS-Aided Communication Systems under Nakagami-*m* Fading Channels

**DOI:** 10.3390/s24113332

**Published:** 2024-05-23

**Authors:** Ricardo Coelho Ferreira, Gustavo Fraidenraich, Felipe A. P. de Figueiredo, Eduardo Rodrigues de Lima

**Affiliations:** 1 Department of Communications, Faculty of Electrical and Computer Engineering, State University of Campinas (UNICAMP), Campinas 13083-970, SP, Brazil; gf@decom.fee.unicamp.br (G.F.); felipe.figueiredo@inatel.br (F.A.P.d.F.); 2 Instituto Nacional de Telecomunicações—INATEL, Santa Rita do Sapucaí 37540-000, MG, Brazil; 3 Department of Hardware Design, Instituto de Pesquisas Eldorado, Alan Turing-275, Campinas 13083-898, SP, Brazil; eduardo.lima@eldorado.org.br

**Keywords:** large intelligent surfaces, reflecting surfaces, mobile communications, Nakagami-*m* fading, Mu-MIMO systems

## Abstract

This study evaluates the performance of large intelligent surface (LIS) technology in the context of a multi-user MIMO mobile communication system (Mu-MIMO) proposed for the sixth generation (6G). LIS employs digitally controlled reflectors to enhance Signal-to-Interference plus Noise Ratio (SINR) and establish line of sight (LoS) connectivity in non-LoS environments, improving transmission security. Analytical expressions are derived to assess LIS performance metrics, including distribution parameters, bit error probability, and secrecy outage probability, considering the presence of eavesdroppers and environmental fading. The study highlights the potential of LIS technology to enhance the confidentiality and reliability of digital communication systems in next-generation networks.

## 1. Introduction

Mobile communications face several challenges in delivering signals with a good signal-to-noise ratio (SNR) and high transmission rates to users. One is multipath propagation, which is present mainly in large urban centers, with increasingly larger buildings and many users sharing the spectrum. This study proposes the implementation of strategically positioned reflecting surfaces to enhance the channel SNR and deliver a clearer, less interfered signal.

Large, intelligent surfaces have been one of the biggest innovations in digital communications in recent years. The term has variants such as large reflecting surfaces or intelligent reflecting surfaces. They consist of a grid of reflecting units composed of a metamaterial with a controllable reflection coefficient that can be digitally controlled using an optimization algorithm to strengthen the channel’s LoS.

Zhang et al. [1] presented a cost-effective and energy-efficient solution to enhance signal coverage and improve system capacity in mobile communications. However, large intelligent surfaces face challenges such as multiplicative fading effects arising from composite channels between the LIS and base stations and between the LIS and users. Additionally, the LIS encounters difficulties in enhancing communication systems when a strong direct link is present. Almekhlafi et al. [2] investigated a scenario where a base station transmits signals to multiple users using a single antenna. They developed an algorithm to jointly optimize power allocation to users and phase shifts induced by the LIS. In contrast to other studies that perform optimization processes in a decoupled manner, the authors propose two solutions: one utilizing linear transformation to reduce the number of variables for optimization and another employing an element-wise Karush–Kuhn–Tucker approach to derive closed-form expressions for phase shifts in a multi-user scenario.

Researchers are actively involved in discussions about the sixth generation and beyond the fifth generation (B5G) of mobile communications in several countries [3,4,5,6]. To meet this advancement, the community faces a series of challenges, encompassing algorithmic and mathematical complexities, as well as issues related to hardware and materials [7,8,9,10].

Tataria et al. [11] contributed significantly to addressing the challenges associated with the real-time implementation of LISs. In the same vein, Wang et al. [12] presented a remarkable system proposal employing a reconfigurable smart reflective surface in a MIMO cognitive radio environment. Their approaches strive to optimize the system secrecy rate by jointly optimizing base station transmit beamforming, and RIS-reflected beamforming, considering the complexities of dynamic environments.

Despite being a relatively new topic, there are older references in the LIS literature [13]. The term LIS has gained evidence and prominence and become a relevant bet for the technological industry and also academic research as a promising alternative to improve spectral efficiency (since it can act passively, without energy expenditure), decrease the bit error probability, and allow the erasure of channels for eavesdroppers using beamforming techniques, allowing a more elaborate approach of physical layer security.

The ability of the LIS to adapt to the channel and generate a resulting channel with different statistics allows us to, in a way, think of a controllable and adjusted channel. The channel between the transmitter and the LIS, as well as the channel between the end-user and the LIS, can be generically modeled using the Nakagami-m distribution, with the *m* parameter allowing a generic analysis of the environment’s behavior [14] before considering the presence of the LIS. In addition, it is prudent to consider a possible direct channel between the user and the transmitter and channels between the transmitter and an eavesdropper; without losing the generality, we can consider all these channels as Nakagami-m. However, each one has its parameter *m*; for users close to the base station, the parameter *m* in the Nakagami distribution for the direct channel will be large between the user and the transmitter and may not even need the LIS, whereas a user outside the LoS may have m=1 and fall into the Rayleigh fading scenario (without LoS), where the LIS will create a channel resulting from the composition of all the links that result in a channel with LoS and a lower bit error rate [15].

The literature on mobile communication network optimization has been limited to two-point operations, with some strategies only for the transmitter (a base station) and the receiver. However, LISs changed this reality and created new possibilities to achieve even lower bit error rates, better spectral efficiency, decreased transmission power, and increased SNR. According to Gong et al. [16] the LIS allows modifying the channel characteristics and canceling its phase; in addition to projecting the beam towards the user, the adjustable reflector panels of the LIS can match the signals that reach them, thus enabling a smart radio environment that learns to beat the channel, even when its characteristics change.

Wu et al. [17] developed a framework in the form of a tutorial for implementing the LIS; the authors present the channel model, including the reflectors, discuss hardware aspects and practical issues related to the system deployment, and point out future possibilities for this technology.

Sánchez et al. [18] presented a performance analysis regarding physical layer security (PLS) in an environment assisted by a LIS, considering the possibility of phase errors. The authors assume that it is possible to model an equivalent scalar fading channel including the LIS, as shown by recent work [19] and show that the eavesdropper’s channel is Rayleigh distributed. The fading coefficient is statistically independent of the channel between the transmitter and the legitimate user; they also present the scaling laws for the SNR of the legitimate channel and the eavesdropper concerning the number of LIS reflectors.

Most preliminary articles on LISs present strategies for estimating the channel assisted by the LIS via least squares and other related methods, considering perfect knowledge of the CSI. However, this scenario is not close to reality since they are passive reflectors that intelligently reflect the inside electromagnetic waves to improve system performance. Nadeem et al. [20] present one of the first and most relevant contributions in studying a multi-user system assisted by the LIS assuming an imperfect CSI. The authors use the a priori knowledge of the fading statistics to feed a Bayesian minimum mean squared error (MMSE) estimator for the resulting fading, thus proposing a joint design of the precoder and the power allocation for transmission considering the application of beamforming in the LIS and show the impact of the channel estimation error on the efficiency of the designed system.

Basar et al. [21] present a script for the analytical calculation of the symbol error probability (SEP) in the transmission through the LIS in a generic scenario, in addition to presenting alternative modeling that considers the LIS as an access point (AP), which can or cannot know the phases of the channel.

The statistical analysis of cascaded MIMO channels poses a considerable challenge, complicating the modeling of resulting fading due to including both linear and non-linear transformations of random variables in its definition. However, by using the central limit theorem (CLT), it is possible to obtain, with great accuracy, the fading statistical model, taking into account the continuous phase errors committed by the LIS after the phase adjustment. The composite channel is the product of two channels with a Nakagami-*m* [14] distribution with different coefficients, modeling what goes to the LIS and what goes to the user, and a complex phase error modeled as a Von Mises distribution. Ferreira et al. [15] obtained the distribution of the resulting channel considering only one antenna in the receiver; in this work, we approach a more complex and more comprehensive system model, which includes all the analyses made by the authors, for a special case in which the number of antennas of the user is unitary.

This study investigates the fading distribution of a LIS-aided channel with multiple transmitters and users enabling the increase of SINR, allowing the strengthening of a LoS or improving spectrum sharing. It examines the impact of channel parameters (the number of LIS reflectors, users, the Von Mises error concentration parameter, and the number of transmitter antennas) on the bit error probability and the secrecy outage probability. The aim is to assess the impact of LIS design on performance and help design the system based on quality and information security metrics, evaluating performance that can be calculated directly through simple algebraic expressions involving the parameters of the statistical modeling of the system.

Recent papers on LISs present various mobile system modeling schemas. Lin et al. [22] focused on joint beamforming design for LIS-aided hybrid satellite–terrestrial relay networks, employing a refracting LIS approach to enhance satellite signals for obstructed users. Lin et al. [23] investigated destructive beamforming by a malicious LIS in IoT networks to minimize received SNR at IoT devices. Ma et al.’s [24] study converted mmWave communications with finite blocklength, optimizing antenna gain for covert transmission against spatially random wardens. Niu et al. [25] analyzed robust beamforming design in a secrecy MISO network with LIS, addressing imperfections in CSI and potential eavesdroppers. These articles considered different beamforming strategies, addressing challenges such as power optimization, security, and robustness to channel variations. In contrast, our paper focuses on the phase error distribution without being restricted to a specific optimization technique and can be applied, regardless of how the LIS is designed, provided that the phase error concentration parameter is known. With this level of abstraction, we analyzed the system performance in terms of physical layer security and bit error probability across various fading scenarios involving multiple users.

## 2. System Model

**Figure 1:**
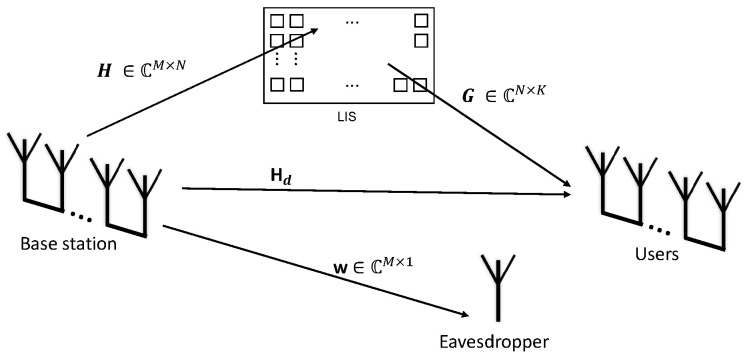
System model with an eavesdropper link.

This work considers a base station composed of *M* uncorrelated antennas and *K* uncorrelated users, as shown in Figure 1. The base station sends the same message to all users; however, it applies a normalized precoding vector for each one of the fading channels.

The signal received by the uncorrelated antenna array at the user side is

(1) $$y=\left(G^{H}\Phi^{H}H^{H}+H_{d}^{H}\right)\Psi +\eta ,$$

where $H\in C^{M\times N}$ is the channel between each one of the uncorrelated antennas at the base station and each one of the LIS reflectors, $G\in C^{N\times K}$ is the channel between the LIS reflectors and the users, $\Phi \in C^{N\times N}$ is the phase matrix with the phase shifts applied by the LIS in the incoming signals, and $H_{d}\in C^{M\times K}$ is the direct link between the base station and each user. The eavesdropper link is $w\in C^{M\times 1}$ and is only considered for the secrecy outage probability analysis. Therefore, it was neglected in the first analysis.

One possible strategy to cancel the channel phase is to apply a precoding matrix at the base station; in this case, the sub-optimal solution is the normalized hermitian of the overall channel.

Let

(2) $$\Upsilon =H\Phi G+H_{d},$$

whose dimensions are $\Upsilon \in C^{M\times K}$, and the elements of the matrix Υ are the $\upsilon_{k}$ vectors.

The scalar elements of H have a Nakagami distribution with parameters $m_{H}$ and $\Omega_{H}$, the elements of G have a Nakagami distribution with parameters $m_{G}$ and $\Omega_{G}$, and the elements of $H_{d}$ follow the complex normal distribution with mean zero.

The mean of a variable *T* with Nakagami-*m* distribution with parameters *m* and Ω can be calculated by

(3) $$E\left[T\right]=\frac{\Gamma \left(m+\frac{1}{2}\right)}{\Gamma (m)}{\left(\frac{\Omega }{m}\right)}^{\frac{1}{2}},$$

and the second-order moment $E\left[T^{2}\right]=\Omega$.

The transmitted symbols after the application of the precoder are

(4) Ψ=PAs,

where *P* is the power gain and *A* is the precoding matrix, and, for analysis purposes, the s symbols are generated as complex normal distributed $s∼CN\left(0,I_{K}\right)$.

The sub-optimal precoding matrix, considering complete knowledge of the Channel State Information (CSI), is given by

(5) $$A=\sqrt{P}\frac{\Upsilon }{\Upsilon_{F}},$$

where $._{F}$ is the Frobenius norm. The precoding vector for each user is given by

(6) $$a_{k}=\sqrt{\frac{P}{K}}\frac{\upsilon_{k}}{\upsilon_{k}},$$

and each one of the $\upsilon_{k}$ has dimensions $\upsilon_{k}\in C^{M\times 1}$.

The scalar definition of each element of the $\upsilon_{k}$ vectors is

(7) $$\upsilon_{lk}=\sum_{p=1}^{N}\sum_{q=1}^{N}h_{lp}\phi_{pq}g_{qk}+h_{(d),kl},$$

after the application of the LIS, these coefficients can be rewritten as

(8) $$\upsilon_{lk}=\sum_{p=1}^{N}\sum_{q=1}^{N}\left|h_{lp}\right|\left|g_{qk}\right|e^{j\left(\delta_{(h),lp}+\delta_{(g),qk}-\varphi_{pq}\right)}+h_{(d),kl},$$

where $\delta_{(h),lp}=arg\left(h\right)$, $\varphi_{pq}=arg\left(\phi_{pq}\right)$, and $\delta_{(g),qk}=arg\left(g_{qk}\right)$ are the phases of each channel.

To nullify the overall channel phase, the phase shift applied by the LIS panel must be equal to

$$\varphi_{pq}=\delta_{(h),lp}+\delta_{(g),qk}-\delta_{pq}$$

where $\delta_{pq}$ is the residual error of the LIS phase correction.

The elements of the overall channel matrix for each user are

(9) $$\upsilon_{k}=\sum_{p=1}^{N}\sum_{q=1}^{N}\left|h_{p}\right|\left|g_{qk}\right|e^{j\delta_{pq}}+h_{(d),k},$$

where

$$h_{p}=\left[h_{1p},h_{2p}\ldots h_{Mp}\right]$$

and $h_{(d),k}=\left[h_{(d),k1},h_{(d),k2},\ldots h_{(d),kM}\right]$ are the lines of the matrix $H_{d}$.

The norm of the overall channel for each user can be written as

(10) $${\upsilon_{k}}^{2}=\sum_{l=1}^{M}{\left|\sum_{p=1}^{N}\sum_{q=1}^{N}\left|h_{lp}\right|\left|g_{qk}\right|e^{j\delta_{pq}}+h_{(d),kl}\right|}^{2},$$

and the SINR at the user antenna *k* is

(11) $$\gamma_{k}=\frac{|\upsilon_{k}^{H}a_{k}{|}^{2}}{\sum_{i=0,i\neq k}^{K}{\left|\upsilon_{i}^{H}a_{i}\right|}^{2}+\sigma_{\eta }^{2}},$$

where the terms

(12) $${\left|\upsilon_{k}^{H}a_{k}\right|}^{2}=\frac{P}{K}{\upsilon_{k}}^{2}.$$

Therefore,

(13) $$\gamma_{k}=\frac{P}{K}\frac{{\upsilon_{k}}^{2}}{\frac{P}{K}\sum_{i=0,i\neq k}^{K}{\upsilon_{i}}^{2}+\sigma_{\eta }^{2}}.$$

The SINR can be rewritten as

(14) $$\gamma_{k}=\frac{Z_{k}}{\sigma_{\eta }^{2}+\sum_{i=0,i\neq k}^{K}Z_{i}},$$

where $Z_{i}={\upsilon_{i}}^{2}$.

Since the summation terms of Equation (10) are complex variables, then

(15) $$Z_{k}=\frac{P}{K}\sum_{l=1}^{M}{\left|r_{lk}\right|}^{2},$$

where

(16) $$r_{lk}=\sum_{p=1}^{N}\sum_{q=1}^{N}\left|h_{lp}\right|\left|g_{qk}\right|e^{j\delta_{pq}}+h_{(d),kl}.$$

Considering that the complex number $r_{lk}=c_{lk}+js_{lk}$, thus

(17) $$Z_{k}=\frac{P}{K}\sum_{l=1}^{M}{\left|c_{lk}+js_{lk}\right|}^{2},$$

in which

(18) $$c_{lk}=\sum_{p=1}^{N}\sum_{q=1}^{N}\left|h_{lp}\right|\left|g_{qk}\right|\cos\delta_{pq}+ℜ\left\{h_{(d),kl}\right\},$$

and

(19) $$s_{lk}=\sum_{p=1}^{N}\sum_{q=1}^{N}\left|h_{lp}\right|\left|g_{qk}\right|\sin\delta_{pq}+ℑ\left\{h_{(d),kl}\right\}.$$

By substituting the imaginary and real parts of the complex scalar $r_{lk}$, it follows that

(20) $${\upsilon_{k}}^{2}=\sum_{l=1}^{M}c_{lk}^{2}+\sum_{l=1}^{M}s_{lk}^{2},$$

since the real and imaginary parts of the direct path $h_{(d),kl}$ are Gaussian and uncorrelated.

Let be $C_{k}=\sum_{l=1}^{M}c_{lk}^{2}$ and $S_{k}=\sum_{l=1}^{M}s_{lk}^{2}$; therefore, we can rewrite ${\upsilon_{k}}^{2}$ as

(21) $$R_{k}={\upsilon_{k}}^{2}=C_{k}+S_{k},$$

then

(22) $$Z_{k}=\frac{P}{K}R_{k},$$

we consider that

(23) $$F_{k}=\sigma_{\eta }^{2}+\sum_{i=0,i\neq k}^{K}Z_{i}.$$

Therefore, the SINR can be written in terms of these two coefficients as

(24) $$\gamma_{k}=\frac{Z_{k}}{F_{k}}.$$

Since $Z_{k}$ is the sum of squared Gaussian random variables, it is reasonable to assume that $Z_{k}$ is Gamma distributed with parameters $\alpha_{Z}$ and $\beta_{Z}$. The term $F_{k}$ is the sum of Gamma random variables, and it is supposed to be also Gamma distributed with parameters $\alpha_{F}$ and $\beta_{F}$.

The SINR $\gamma_{k}$ is the ratio between the variables $Z_{k}$ and $F_{k}$ and also is supposed to be Gamma distributed. This study proposes an approximation for the SINR distribution to obtain the error and secrecy outage probability. Although not an exact solution, the approximation is very accurate, even for small values of *M*, *N*, and *K*.

The parameters of the SINR distribution can be obtained by calculating the moments of $\gamma_{k}$. Since $\gamma_{k}$ is the ratio of $Z_{k}$ and $F_{k}$, therefore, the statistics of the numerator $Z_{k}$ and the denominator $F_{k}$ must be evaluated.

The variables $Z_{k}$ and $F_{k}$ are correlated, and because of this, the covariance between them must be taken into account.

According to Kendall et al. [26], the mean of the ratio between the Gamma random variables is

(25) $$\mu_{\gamma }=E\left[\gamma_{k}\right]=\frac{\mu_{Z}}{\mu_{F}},$$

where $\mu_{Z}=E\left[Z_{k}\right]$ and $\mu_{F}=E\left[F_{k}\right]$.

The variance can be approximated by

(26) $$var\left(\gamma_{k}\right)={\left(\frac{\mu_{Z}}{\mu_{F}}\right)}^{2}\left[\frac{\sigma_{Z}^{2}}{\mu_{Z}^{2}}-2\left(\frac{cov(Z_{k},F_{k})}{\mu_{Z}\mu_{F}}\right)+\frac{\sigma_{F}^{2}}{\mu_{F}^{2}}\right],$$

where $\sigma_{F}^{2}=var\left(F_{k}\right)$ and $\sigma_{Z}^{2}=var\left(Z_{k}\right)$.

The term $cov\left(Z_{k},F_{k}\right)=E\left[Z_{k}F_{k}\right]-E\left[Z_{k}\right]E\left[F_{k}\right]$ can be computed as $cov(Z_{k},F_{k})=$$E\left[Z_{k}\sum_{i\neq k}^{K}Z_{i}\right]-E\left[Z_{k}\right]E\left[\sum_{i\neq k}^{K}Z_{i}\right]=\sum_{i\neq k}^{K}\left(E\left[Z_{k}Z_{i}\right]-E\left[Z_{k}\right]E\left[Z_{i}\right]\right)$; by considering the definition of the covariance and considering that all the coefficients $Z_{k}$ are equally distributed, then

(27) $$cov\left(Z_{k},F_{k}\right)=\left(K-1\right)cov\left(Z_{i},Z_{k}\right),\forall i\neq k.$$

Given the covariance $cov(Z_{k},F_{k})$, the variance of $\gamma_{k}$ can be derived as

(28) $$\sigma_{\gamma }^{2}={\left(\frac{\mu_{Z}}{\mu_{F}}\right)}^{2}\left[\frac{\sigma_{Z}^{2}}{\mu_{Z}^{2}}-2\left(\frac{\left(K-1\right)cov\left(Z_{i},Z_{k}\right)}{\mu_{Z}\mu_{F}}\right)+\frac{\sigma_{F}^{2}}{\mu_{F}^{2}}\right],$$

where $\sigma_{\gamma }^{2}$ is an approximation for $var\left(\gamma_{k}\right)$.

Since $\mu_{F}=\left(K-1\right)\mu_{Z}$, thus

(29) $$\sigma_{\gamma }^{2}=\frac{1}{{\left(K-1\right)}^{2}\mu_{Z}^{2}}\left[\sigma_{Z}^{2}-2cov\left(Z_{i},Z_{k}\right)+\frac{\sigma_{F}^{2}}{{\left(K-1\right)}^{2}}\right],$$

with the overall channel moments, the fading parameters $\alpha_{\gamma }$ and $\beta_{\gamma }$ can be computed as follows:

(30) $$\alpha_{\gamma }=\frac{\mu_{\gamma }^{2}}{\sigma_{\gamma }^{2}},\;\;\beta_{\gamma }=\frac{\mu_{\gamma }}{\sigma_{\gamma }^{2}},$$

where $\alpha_{\gamma }$ and $\beta_{\gamma }$ are the shape and rate parameters of the SINR $\gamma_{k}$.

The mean and variance of $Z_{k}$, $F_{k}$ and $\gamma_{k}$ are presented in Appendix A, Appendix B and Appendix C respectively.

### 2.1. Error Probability

The error probability of a LIS-aided communication system was derived by Ferreira et al. [19], considering that the transmitted symbols are *M*-QAM signals transmitted through a Gamma fading channel; in this scenario, the probability will be

(31) $${\bar{P}}_{e}^{QAM}\left(\gamma \right)\approx \frac{4}{\log_{2}M}Q\left(\sqrt{\frac{3\gamma {log}_{2}M}{M-1}}\right).$$

Since the mu-MIMO LIS channel is considered as Gamma distributed, therefore, this expression for ${\bar{P}}_{e}^{QAM}\left(\gamma \right)$ will be used. The parameters $\gamma_{k}$ are identically distributed and, therefore, ${\bar{P}}_{e}^{QAM}\left(\gamma_{k}\right)={\bar{P}}_{e}^{QAM}\left(\gamma_{l}\right)={\bar{P}}_{e}^{QAM}\left(\gamma \right)$.

### 2.2. Secrecy Outage Probability

Secrecy outage probability is a metric used in communication systems to quantify the likelihood of unauthorized information disclosure. It represents the probability that the confidential information transmitted between parties becomes susceptible to interception or eavesdropping. A lower secrecy outage probability indicates a higher level of security, where the confidential information is less likely to be compromised during transmission.

The SOP is the probability that the instantaneous secrecy capacity, *C*, is less or equal to a given capacity threshold, $\ln\left(1+\gamma_{th}\right)$, which is expressed as

(32) $$SOP=Pr\left[\ln\frac{\left(1+\gamma_{D}\right)}{\left(1+\gamma_{E}\right)}\leq \ln\left(1+\gamma_{th}\right)\right]=\int_{0}^{\infty }\int_{0}^{\left(1+\gamma_{E}\right)\left(1+\gamma_{D}\right)-1}f_{\gamma_{E}}\left(w\right)f_{\gamma_{D}}\left(u\right)dudw,$$

the instantaneous secrecy capacity can be written as

(33) $$C=\left\{\begin{array}{cc} \ln\left(1+\gamma_{D}\right)-\ln\left(1+\gamma_{E}\right) & \gamma_{D}>\gamma_{E}\\ 0 & \gamma_{D}\leq \gamma_{E} \end{array}\right.,$$

where $\gamma_{E}$ is the SINR of the channel between the source and the eavesdropper and $\gamma_{D}$ is the SINR of the channel between the source and the correct destination (the user).

Ferreira et al. [15] derived the exact formula of the SOP for a Nakagami-*m* distributed eavesdropper channel as (34).

(34) $$\begin{array}{c} SOP=\sum_{k=0}^{\infty }\frac{{\left(-1\right)}^{k}\beta^{\alpha +k}\gamma_{th}^{\alpha +k}}{\Gamma (\alpha )\Gamma (k+1)}\\ \times (\frac{\pi m^{m}2^{-\alpha -k}v^{-2m}\Omega^{-m}\Gamma \left(m+\frac{1}{2}\right)\csc{\left(\pi \left(\alpha +k+2m\right)\right)}_{2}{\tilde{F}}_{2}\left(m,m+\frac{1}{2};\frac{1}{2}\left(k+2m+\alpha +1\right),\frac{1}{2}\left(k+2m+\alpha +2\right);-\frac{m}{v^{2}\Omega }\right)}{\Gamma (-k-\alpha +1)}\\ +\frac{\pi^{3/2}\Omega^{\frac{\alpha +k}{2}}m^{\frac{1}{2}\left(-\alpha -k\right)}v^{\alpha +k}}{2\Gamma (m)}(\frac{2\csc{\left(\frac{1}{2}\pi \left(\alpha +k+2m\right)\right)}_{2}{\tilde{F}}_{2}\left(\frac{1}{2}\left(-k-\alpha \right),\frac{1}{2}\left(-k-\alpha +1\right);\frac{1}{2},\frac{1}{2}\left(-k-2m-\alpha +2\right);-\frac{m}{v^{2}\Omega }\right)}{\alpha +k}\\ -\frac{\sqrt{m}\sec{\left(\frac{1}{2}\pi \left(\alpha +k+2m\right)\right)}_{2}{\tilde{F}}_{2}\left(\frac{1}{2}\left(-k-\alpha +1\right),\frac{1}{2}\left(-k-\alpha +2\right);\frac{3}{2},\frac{1}{2}\left(-k-2m-\alpha +3\right);-\frac{m}{v^{2}\Omega }\right)}{v\sqrt{\Omega }})), \end{array}$$

where $v=\frac{1+\gamma_{th}}{\gamma_{th}}$.

## 3. Numerical Results

This section demonstrates the performance of the LIS for a multi-user system through simulations using the Monte Carlo method and analyzing the validity of the proposed approximations for various analysis scenarios in terms of the number of antennas at the LIS panel, the number of users in the system, the number of antennas at the base station, and the Von Mises parameter for the phase error distribution.

In this study, the SINR was simulated for each user using the parameters of each channel involved in the total link, being the Nakagami-*m* channels from the base station to the LIS, from the LIS to the user, and the complex normal direct channel from the base station to the user, which may be strong for near-field communications and weak for far-field communications. The SINR histograms were generated by the Monte Carlo method, simulating the resulting channel several times, and the contours of the histograms are shown close to the PDF of the Gamma distribution.

### 3.1. Probability Distribution

**Figure 2:**
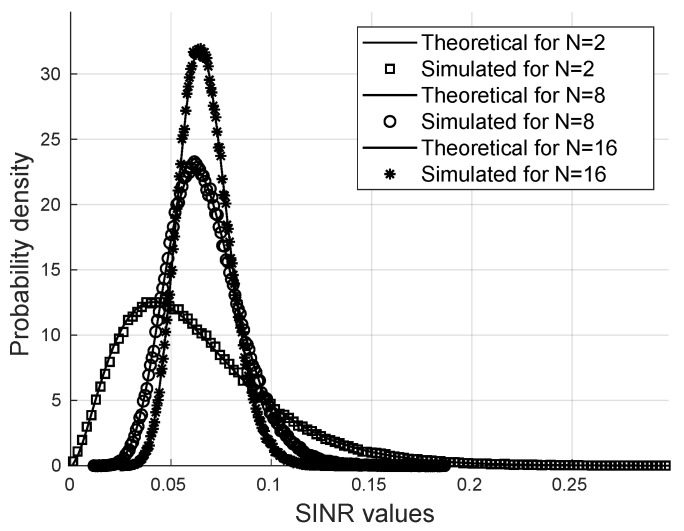
Probability distribution function via Monte Carlo.

For $10^{5}$ iterations of the Monte Carlo method, it is shown in Figure 2 that the probability density function of the SINR is close to the Gamma distribution.

Figure 2 shows a comparison between the exact Gamma distribution and the simulated PDF of the SINR via the Monte Carlo method for m=2,Ω=1,K=16 users, and M=2 for different values of the number of reflectors *N*.

It is possible to note that the SINR follows a gamma distribution for a wide variety of parameters in such a way that the analytical calculations of the moments and the statistical parameters of the SINR are valid for the formulas proposed for the probability of bit error and the secrecy outage probability.

### 3.2. Bit Error Probability

One possible way to demonstrate that the LIS can improve the SINR is by showing how the bit error probability varies, increasing the number of antennas at the base station, the number of reflecting elements, and the concentration parameter of the phase error distribution.

**Figure 3:**
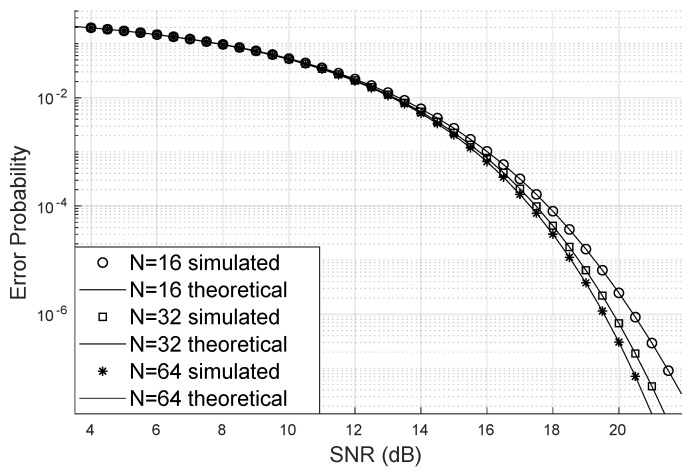
Error probability varying with *N*.

Figure 3 illustrates how the bit error probability decreases when the number of reflectors increases. This scenario has four users, eight transmitting antennas, the Nakagami parameter m=2, and the Von Mises κ=2. Notably, although the bit error probability is low, the values do not change significantly from N=32 to N=64 as much as from N=16 to N=32 reflectors. This may be because the channel already has LoS. Additionally, and beyond a certain number of reflectors, the SINR becomes optimal with the assistance of the LIS, which is something this type of analysis can help discover.

**Figure 4:**
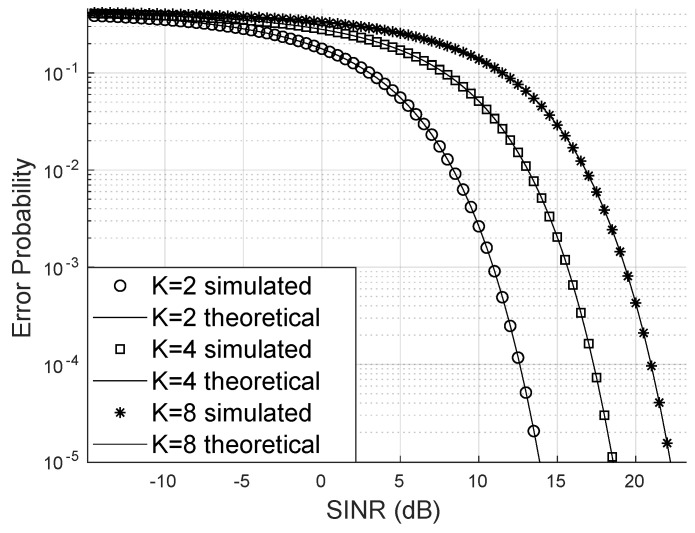
Error probability for m=2, 16-QAM.

Figure 4 demonstrates that increasing the number of system users raises the bit error probability when keeping the number of LIS reflectors, base station antennas, and the Von Mises parameter constant. In this scenario, the transmission of 16-QAM signals was considered. One strategy to mitigate the increase of the error probability is to use many reflectors to enhance the LIS’s capacity to serve more users with higher SINR and, consequently, reduce the bit error probability.

**Figure 5:**
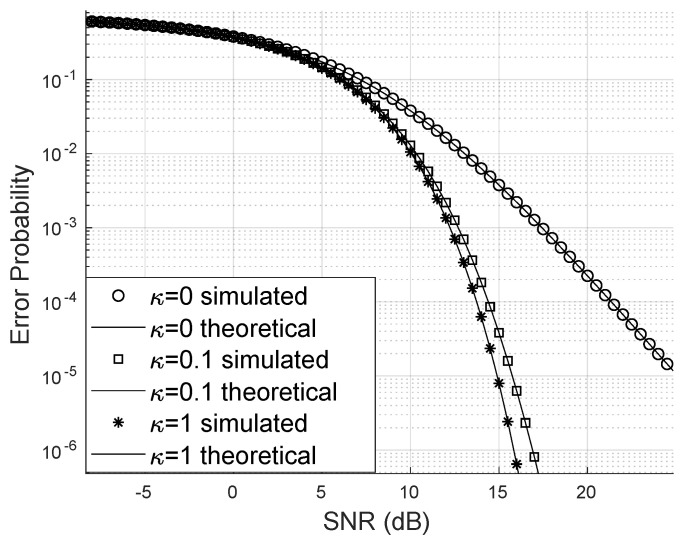
Error probability for different κ values.

For the scenario depicted in Figure 5, in which signals from the 4-QAM constellation are transmitted, the approximation of SINR by the gamma distribution continues to hold, and the bit error probability remains higher when the system accommodates more users.

**Figure 6:**
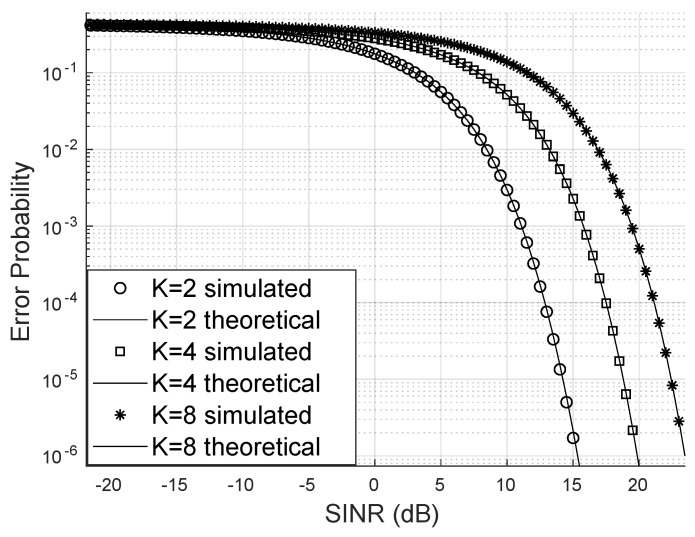
Error probability for m=1 (Rayleigh), 16-QAM.

**Figure 7:**
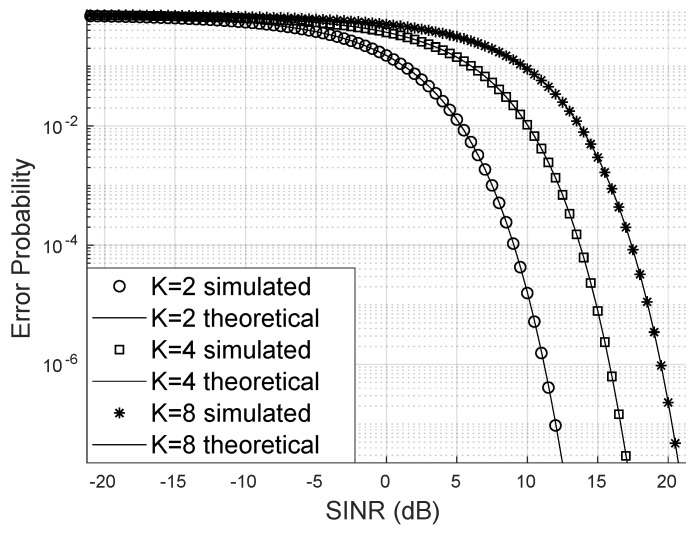
Error probability for m=2, 4-QAM.

When there is no line of sight as in Figure 6, where the scenario with SINR channels following a Rayleigh distribution (Nakagami with m=1) is analyzed, it is noticeable that the exact bit error probability continues to approximate the simulated bit error rate closely. In this case, a higher SINR was required compared to Figure 7 to reduce the bit error rate promoted by the LIS. In this simulation, the number of reflectors was kept constant. However, it is worth noting that one case deals with the 4-QAM constellation and the other with the 16-QAM constellation.

Regarding the Von Mises parameter, it is notable that the bit error probability decreases faster for larger values of κ, as observed in Figure 5. The worst-case scenario is when κ=0 and the error distribution is uniform. In this case, large and small phase errors have the same probability density and occur when the phase error has been poorly corrected.

If the phase error is concentrated around the mean (in this study, the mean phase error is zero), the PDF of the phase error approaches a delta function at the origin. In this case, the SINR will be higher because the sum of phasors will be greater if their phase is zero, and the complex exponential vanishes, leaving only the magnitudes.

### 3.3. Secrecy Outage Probability

To simulate the secrecy outage probability (SOP), the SINR corrected by the LIS was generated, and an additional eavesdropper channel with a Nakagami distribution with LoS access to the LIS signal was considered. This additional channel has a line of sight. The summation was truncated at index 100 to calculate the SOP, and the approximation closely matched the simulated SOP values via Monte Carlo. It was assumed that the Von Mises concentration parameter was κ=2, and the direct link had unity variance.

**Figure 8:**
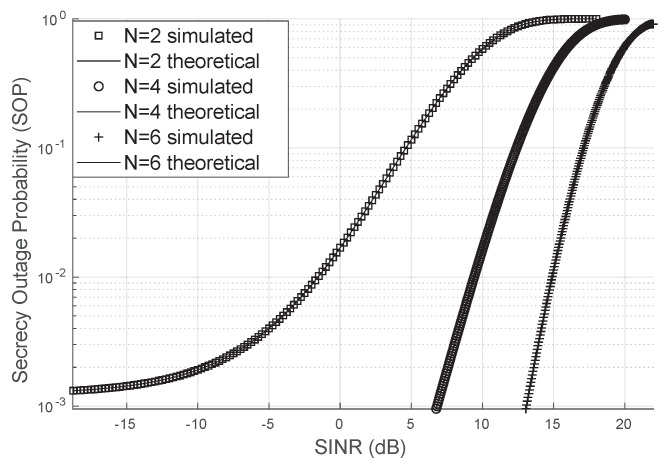
Secrecy Outage Probability.

It can be observed in Figure 8, where an eavesdropper channel with m=2 and Ω=1 is considered, that the higher the number of reflectors, the lower the SOP will be. This demonstrates that the LIS contributes to the confidentiality of information.

## 4. Conclusions

This study shows that Mu-MIMO channels formed by links with Nakagami-*m* fading, when assisted by large intelligent surfaces, have an SINR with Gamma distribution and can considerably reduce their bit error probability by increasing the number of reflectors or the efficiency of the phase optimization algorithm (related to the κ parameter of the phase error distribution).

The worst case, considering the Nakagami-*m* fading channel, is the Rayleigh fading (for m=1); this study shows that Rayleigh channels can be converted to Gamma fading channels by applying the phase shift matrix of the LIS. It was also shown that creating a line of sight in scenarios where a LoS did not exist was possible. This study presented the probability distributions of SINR compared to the PDF of the Gamma distribution. It showed that the exact formula for the probability density is very close to the one generated by the histogram obtained from the channel simulation.

It was possible to show that the LIS can enable a low secrecy outage probability, even for a few reflecting units and low bit error probability values, when increasing the number of LIS elements or improving the quality of phase correction. Our study assumes complete knowledge of the channel state information. It considers phase errors due to the behavior of the reflecting panel when attempting to correct the phase of many signals simultaneously (and not being able to optimize this for all) or due to the influence of the superposition of electromagnetic signals.

## Data Availability

Data are contained within the article.

## Appendix A. Statistical Parameters of *Z_k_*

### Appendix A.1. Expected Value of Z_k_

The expected value of $Z_{k}$ will be

(A1) $$E\left[Z_{k}\right]=\frac{P}{K}E\left[\sum_{l=1}^{M}{\left|r_{lk}\right|}^{2}\right],$$

since the expected value is a linear operator, then

(A2) $$E\left[Z_{k}\right]=\frac{P}{K}\sum_{l=1}^{M}E\left[{\left|r_{lk}\right|}^{2}\right]=\frac{PM}{K}E\left[{\left|r_{lk}\right|}^{2}\right],$$

where the term $E\left[{\left|r_{lk}\right|}^{2}\right]$ must be calculated, but, first of all, the terms $E\left[c_{lk}\right]$ and $E\left[s_{lk}\right]$ are needed.

The expected value $E\left[c_{lk}\right]$ of the real part of $r_{lk}$ can be defined as

(A3) $$E\left[c_{lk}\right]=E\left[\sum_{p=1}^{N}\sum_{q=1}^{N}\left|h_{lp}\right|\left|g_{qk}\right|\cos\delta_{pq}+ℜ\left\{h_{(d),kl}\right\}\right],$$

since $E\left[ℜ\{h_{(d),kl}\}\right]=0$ and the summation terms are independent and equally likely. Therefore,

(A4) $$E\left[c_{lk}\right]=N^{2}E\left[\left|h_{lp}\right|\right]E\left[\left|g_{qk}\right|\right]E\left[\cos\delta_{pq}\right]=N^{2}\mu_{h}\mu_{g}\alpha_{1}.$$

The moments of the fading channels are $\mu_{h}=E\left[\left|h_{lp}\right|\right]$, $\xi_{h}=E\left[{\left|h_{lp}\right|}^{2}\right]$, $\mu_{q}=E\left[\left|g_{qk}\right|\right]$, $\xi_{g}=E\left[{\left|g_{qk}\right|}^{2}\right]$, $\chi_{h}=E\left[{\left|h_{lp}\right|}^{3}\right]$, $\chi_{g}=E\left[{\left|g_{qk}\right|}^{3}\right]$, $\epsilon_{h}=E\left[{\left|h_{lp}\right|}^{4}\right]$, $\epsilon_{g}=E\left[{\left|g_{qk}\right|}^{4}\right]$, $\mu_{ℜ\{h_{\left(d\right)}\}}=E\left[ℜ\{h_{(d),kl}\}\right]=0$, $\xi_{ℜ\{h_{\left(d\right)}\}}=E\left[ℜ{\left\{h_{(d),kl}\right\}}^{2}\right]$, $\mu_{ℑ\{h_{\left(d\right)}\}}=E\left[ℑ\{h_{(d),kl}\}\right]=0$, and $\xi_{ℑ\{h_{\left(d\right)}\}}=E\left[ℑ{\left\{h_{(d),kl}\right\}}^{2}\right]$ and most of then will appear in the following equations.

The expected value $E\left[s_{lk}\right]$ of the imaginary part of $r_{lk}$ can be computed as

(A5) $$E\left[s_{lk}\right]=E\left[\sum_{p=1}^{N}\sum_{q=1}^{N}\left|h_{lp}\right|\left|g_{qk}\right|\sin\delta_{pq}+ℑ\left\{h_{(d),kl}\right\}\right],$$

since $E\left[ℑ\{h_{(d),kl}\}\right]=0$ and the summation terms are independent and equally likely, then

(A6) $$E\left[s_{lk}\right]=N^{2}E\left[\left|h_{lp}\right|\right]E\left[\left|g_{qk}\right|\right]E\left[\sin\delta_{pq}\right]=N^{2}\mu_{h}\mu_{g}\beta_{1},$$

but the Von Mises variable δ has a PDF that is symmetric about zero, and the phase errors are zero mean, so $\forall p.\beta_{p}=E\left[\sin p\delta \right]=0$, therefore

(A7) $$E\left[s_{lk}\right]=N^{2}\mu_{h}\mu_{g}\beta_{1}=0.$$

The expected value $E\left[{\left|r_{lk}\right|}^{2}\right]$ of the magnitude of $r_{lk}$ can be obtained as

(A8) $$E\left[{\left|r_{lk}\right|}^{2}\right]=E\left[c_{lk}^{2}\right]+E\left[s_{lk}^{2}\right],$$

where

(A9) $$E\left[c_{lk}^{2}\right]=E\left[{\left(\sum_{p=1}^{N}\sum_{q=1}^{N}\left|h_{lp}\right|\left|g_{qk}\right|\cos\delta_{pq}+ℜ\left\{h_{(d),kl}\right\}\right)}^{2}\right],$$

and

(A10) $$E\left[s_{lk}^{2}\right]=E\left[{\left(\sum_{p=1}^{N}\sum_{q=1}^{N}\left|h_{lp}\right|\left|g_{qk}\right|\sin\delta_{pq}+ℑ\left\{h_{(d),kl}\right\}\right)}^{2}\right],$$

by expanding the terms (A9), it follows that

(A11) $$\begin{array}{c} E\left[c_{lk}^{2}\right]=E\left[\left(\sum_{p=1}^{N}\sum_{q=1}^{N}\left|h_{lp}\right|\left|g_{qk}\right|\cos\delta_{pq}+ℜ\left\{h_{(d),kl}\right\}\right)\times \ldots \right.\\ \;\left.\left(\sum_{b=1}^{N}\sum_{e=1}^{N}\left|h_{lb}\right|\left|g_{ek}\right|\cos\delta_{be}+ℜ\left\{h_{(d),kl}\right\}\right)\right], \end{array}$$

that can be rewritten as

(A12) $$\begin{array}{c} E\left[c_{lk}^{2}\right]=E\left[\sum_{p=1}^{N}\sum_{q=1}^{N}\sum_{p=1}^{N}\sum_{q=1}^{N}\left(\left|h_{lp}\right|\left|g_{qk}\right|\cos\delta_{pq}\right)\left(\left|h_{lp}\right|\left|g_{qk}\right|\cos\delta_{pq}\right)\right]+E\left[ℜ{\left\{h_{(d),kl}\right\}}^{2}\right]+\ldots \\ \;E\left[ℜ\left\{h_{(d),kl}\right\}\sum_{p=1}^{N}\sum_{q=1}^{N}\left|h_{lp}\right|\left|g_{qk}\right|\cos\delta_{pq}\right]+E\left[ℜ\left\{h_{(d),kl}\right\}\sum_{b=1}^{N}\sum_{e=1}^{N}\left|h_{lb}\right|\left|g_{ek}\right|\cos\delta_{be}\right], \end{array}$$

and since

(A13) $$E\left[ℜ\left\{h_{(d),kl}\right\}\sum_{p=1}^{N}\sum_{q=1}^{N}\left|h_{lp}\right|\left|g_{qk}\right|\cos\delta_{pq}\right]=E\left[ℜ\left\{h_{(d),kl}\right\}\sum_{b=1}^{N}\sum_{e=1}^{N}\left|h_{lb}\right|\left|g_{ek}\right|\cos\delta_{be}\right],$$

and the expected value is a linear operator, so

(A14) $$\begin{array}{c} E\left[c_{lk}^{2}\right]=\sum_{p=1}^{N}\sum_{q=1}^{N}\sum_{b=1}^{N}\sum_{e=1}^{N}E\left[\left|h_{lp}\right|\left|g_{qk}\right|\left|h_{lb}\right|\left|g_{ek}\right|\cos\delta_{pq}\cos\delta_{be}\right]+E\left[ℜ{\left\{h_{(d),kl}\right\}}^{2}\right]+\ldots \\ \;2E\left[ℜ\{h_{(d),kl}\}\right]\sum_{b=1}^{N}\sum_{e=1}^{N}E\left[\left|h_{lb}\right|\left|g_{ek}\right|\cos\delta_{be}\right], \end{array}$$

by separating the independent coefficients, it follows that

(A15) $$\begin{array}{c} E\left[c_{lk}^{2}\right]=\sum_{p=1}^{N}\sum_{q=1}^{N}\sum_{b=1}^{N}\sum_{e=1}^{N}E\left[\left|h_{lp}\right|\left|h_{lb}\right|\right]E\left[\left|g_{qk}\right|\left|g_{ek}\right|\right]E\left[\cos\delta_{pq}\cos\delta_{be}\right]+E\left[ℜ{\left\{h_{(d),kl}\right\}}^{2}\right]+\\ \;2E\left[ℜ\{h_{(d),kl}\}\right]\sum_{b=1}^{N}\sum_{e=1}^{N}E\left[\left|h_{lb}\right|\right]E\left[\left|g_{ek}\right|\right]E\left[\cos\delta_{be}\right]. \end{array}$$

To compute $E\left[c_{lk}^{2}\right]$, four situations must be considered: $N^{2}$ times the event p=b and q=e will occur in the summation, $N^{2}\left(N-1\right)$ times the event p=b and q≠e will occur, $N^{2}{\left(N-1\right)}^{2}$ times p≠b and q≠e, and $N^{2}\left(N-1\right)$ times p≠b and q=e, then

(A16) $$\begin{array}{c} E\left[{\left(\sum_{p=1}^{N}\sum_{q=1}^{N}\left|h_{lp}\right|\left|g_{qk}\right|\cos\delta_{pq}+ℜ\left\{h_{(d),kl}\right\}\right)}^{2}\right]=N^{2}{\left(N-1\right)}^{2}\left({\left(E\left[\left|g_{qk}\right|\right]\right)}^{2}{\left(E\left[\cos\delta_{pq}\right]\right)}^{2}\right)\\ \;+N^{2}\left(E\left[{\left|h_{lp}\right|}^{2}\right]E\left[{\left|g_{qk}\right|}^{2}\right]E\left[\cos^{2}\delta_{pq}\right]\right)+N^{2}\left(N-1\right)\left(E\left[{\left|h_{lp}\right|}^{2}\right]{\left(E\left[\left|g_{qk}\right|\right]\right)}^{2}{\left(E\left[\cos\delta_{pq}\right]\right)}^{2}\right)\\ \;+N^{2}\left(N-1\right)\left({\left(E\left[\left|h_{lp}\right|\right]\right)}^{2}{\left(E\left[\left|h_{lp}\right|\right]\right)}^{2}E\left[{\left|g_{qk}\right|}^{2}\right]{\left(E\left[\cos\delta_{pq}\right]\right)}^{2}\right)+\\ \;E\left[ℜ{\left\{h_{(d),kl}\right\}}^{2}\right]+2E\left[ℜ\{h_{(d),kl}\}\right]\sum_{b=1}^{N}\sum_{e=1}^{N}E\left[\left|h_{lb}\right|\right]E\left[\left|g_{ek}\right|\right]E\left[\cos\delta_{be}\right]. \end{array}$$

Therefore,

(A17) $$\begin{array}{c} E\left[c_{lk}^{2}\right]=\frac{N^{2}}{2}\xi_{h}\xi_{g}\left(1+\alpha_{2}\right)+N^{2}\left(N-1\right)\xi_{h}\mu_{q}^{2}\alpha_{1}^{2}+N^{2}\left(N-1\right)\left(\mu_{h}^{2}\xi_{g}\alpha_{1}^{2}\right)+\\ \;N^{2}{\left(N-1\right)}^{2}\left(\mu_{h}^{2}\mu_{q}^{2}\alpha_{1}^{2}\right)+2N^{2}\mu_{ℜ\{h_{\left(d\right)}\}}\mu_{h}\mu_{q}\alpha_{1}+\xi_{ℜ\{h_{\left(d\right)}\}}. \end{array}$$

The expected value $E\left[s_{lk}^{2}\right]$ of the squared magnitude of $s_{lk}$ can be calculated as

(A18) $$E\left[s_{lk}^{2}\right]=E\left[{\left(\sum_{p=1}^{N}\sum_{q=1}^{N}\left|h_{lp}\right|\left|g_{qk}\right|\sin\delta_{pq}+ℑ\left\{h_{(d),kl}\right\}\right)}^{2}\right],$$

that can be rewritten as

(A19) $$\begin{array}{c} E\left[s_{lk}^{2}\right]=E\left[\sum_{p=1}^{N}\sum_{q=1}^{N}\sum_{b=1}^{N}\sum_{e=1}^{N}\left(\left|h_{lp}\right|\left|g_{qk}\right|\sin\delta_{pq}\right)\left(\left|h_{lb}\right|\left|g_{ek}\right|\sin\delta_{be}\right)\right]+E\left[ℑ{\left\{h_{(d),kl}\right\}}^{2}\right]+\\ \;E\left[ℑ\left\{h_{(d),kl}\right\}\sum_{p=1}^{N}\sum_{q=1}^{N}\left|h_{lp}\right|\left|g_{qk}\right|\sin\delta_{pq}\right]+E\left[ℑ\left\{h_{(d),kl}\right\}\sum_{b=1}^{N}\sum_{e=1}^{N}\left|h_{lb}\right|\left|g_{ek}\right|\sin\delta_{be}\right]. \end{array}$$

After expanding and simplifying the terms, it follows that

(A20) $$\begin{array}{c} E\left[s_{lk}^{2}\right]=\sum_{p=1}^{N}\sum_{q=1}^{N}\sum_{b=1}^{N}\sum_{e=1}^{N}E\left[\left|h_{lp}\right|\left|h_{lb}\right|\right]E\left[\left|g_{qk}\right|\left|g_{ek}\right|\right]E\left[\sin\delta_{pq}\sin\delta_{be}\right]+E\left[ℑ{\left\{h_{(d),kl}\right\}}^{2}\right]+\\ \;2E\left[ℑ\{h_{(d),kl}\}\right]\sum_{b=1}^{N}\sum_{e=1}^{N}E\left[\left|h_{lb}\right|\right]E\left[\left|g_{ek}\right|\right]E\left[\sin\delta_{be}\right], \end{array}$$

that can be simplified to

(A21) $$\begin{array}{c} E\left[s_{lk}^{2}\right]=\frac{N^{2}}{2}\xi_{h}\xi_{g}\left(1-\alpha_{2}\right)+N^{2}\left(N-1\right)\xi_{h}\mu_{q}^{2}\beta_{1}^{2}+N^{2}\left(N-1\right)\left(\mu_{h}^{2}\xi_{g}\beta_{1}^{2}\right)+\\ \;N^{2}{\left(N-1\right)}^{2}\left(\mu_{h}^{2}\mu_{q}^{2}\beta_{1}^{2}\right)+2N^{2}\mu_{ℑ\{h_{\left(d\right)}\}}\mu_{h}\mu_{q}\beta_{1}+\xi_{ℑ\{h_{\left(d\right)}\}}, \end{array}$$

and, since $\beta_{1}=0$, therefore,

(A22) $$E\left[s_{lk}^{2}\right]=\frac{N^{2}}{2}\xi_{h}\xi_{g}\left(1-\alpha_{2}\right)+\xi_{ℑ\{h_{\left(d\right)}\}}.$$

To compute the expected value $E\left[Z_{k}\right]$ substituting (A8) in (A2), then

(A23) $$E\left[Z_{k}\right]=\frac{PM}{K}\left(E\left[c_{lk}^{2}\right]+E\left[s_{lk}^{2}\right]\right).$$

After substituting (A17) and (A22) in (A23), it follows that

(A24) $$\begin{array}{c} \mu_{Z_{k}}=E\left[Z_{k}\right]=\frac{PM}{K}\left(\frac{N^{2}}{2}\xi_{h}\xi_{g}\left(1+\alpha_{2}\right)+N^{2}\left(N-1\right)\xi_{h}\mu_{q}^{2}\alpha_{1}^{2}+N^{2}\left(N-1\right)\left(\mu_{h}^{2}\xi_{g}\alpha_{1}^{2}\right)+\right.\\ \;\left.N^{2}{\left(N-1\right)}^{2}\left(\mu_{h}^{2}\mu_{q}^{2}\alpha_{1}^{2}\right)+2N^{2}\mu_{ℜ\{h_{\left(d\right)}\}}\mu_{h}\mu_{q}\alpha_{1}+\xi_{ℜ\{h_{\left(d\right)}\}}+\frac{N^{2}}{2}\xi_{h}\xi_{g}\left(1-\alpha_{2}\right)+\xi_{ℑ\{h_{\left(d\right)}\}}\right). \end{array}$$

### Appendix A.2. Variance of Z_k_

To compute the variance of $Z_{k}$, we consider that

(A25) $$\sigma_{Z_{k}}^{2}=var\left(Z_{k}\right)=var\left(\frac{P}{K}\sum_{l=1}^{M}{\left|r_{lk}\right|}^{2}\right),$$

in terms of the real and imaginary parts, the equation can be rewritten as

(A26) $$\sigma_{Z_{k}}^{2}={\left(\frac{P}{K}\right)}^{2}var\left(\sum_{l=1}^{M}c_{lk}^{2}+\sum_{l=1}^{M}s_{lk}^{2}\right),$$

since the variance of the sum of random variables can be computed in terms their covariances, then

(A27) $$\sigma_{Z_{k}}^{2}={\left(\frac{P}{K}\right)}^{2}\left[var\left(C_{k}\right)+var\left(S_{k}\right)+2cov\left(C_{k},S_{k}\right)\right],$$

where

(A28) $$var\left(C_{k}\right)=var\left(\sum_{l=1}^{M}c_{lk}^{2}\right)=M\left(M-1\right)cov\left(c_{lk},c_{ik}\right)+Mvar\left(c_{lk}\right),$$

(A29) $$var\left(S_{k}\right)=var\left(\sum_{l=1}^{M}s_{lk}^{2}\right)=M\left(M-1\right)cov\left(s_{lk},s_{ik}\right)+Mvar\left(s_{lk}\right),$$

and

(A30) $$cov\left(C_{k},S_{k}\right)=E\left[C_{k}S_{k}\right]-E\left[C_{k}\right]E\left[S_{k}\right].$$

To compute the previous variances, the covariances $cov\left(c_{lk},c_{ik}\right)$ and $cov\left(s_{lk},s_{ik}\right)$ are needed; we consider that

(A31) $$cov\left(c_{lk},c_{ik}\right)=E\left[c_{lk}c_{ik}\right]-E\left[c_{lk}\right]E\left[c_{ik}\right]\to cov\left(c_{lk},c_{ik}\right)=E\left[c_{lk}c_{ik}\right]-{\left(E\left[c_{lk}\right]\right)}^{2}$$

and

(A32) $$cov\left(s_{lk},s_{ik}\right)=E\left[s_{lk}s_{ik}\right]-E\left[s_{lk}\right]E\left[s_{ik}\right]\to cov\left(s_{lk},s_{ik}\right)=E\left[s_{lk}s_{ik}\right]-{\left(E\left[s_{lk}\right]\right)}^{2},$$

since the expected value $E\left[C_{k}S_{k}\right]$ can be obtained as

(A33) $$\begin{array}{c} E\left[C_{k}S_{k}\right]=E\left[\left(\sum_{l=1}^{M}{\left(\sum_{p=1}^{N}\sum_{q=1}^{N}\left|h_{lp}\right|\left|g_{qk}\right|\cos\delta_{pq}+ℜ\left\{h_{(d),kl}\right\}\right)}^{2}\right)\times \ldots \right.\\ \;\left.\left(\sum_{l=1}^{M}{\left(\sum_{p=1}^{N}\sum_{q=1}^{N}\left|h_{lp}\right|\left|g_{qk}\right|\sin\delta_{pq}+ℑ\left\{h_{(d),kl}\right\}\right)}^{2}\right]\right), \end{array}$$

thus

(A34) $$\begin{array}{c} E\left[c_{lk}c_{ik}\right]=E\left[\left(\sum_{p=1}^{N}\sum_{q=1}^{N}\left|h_{lp}\right|\left|g_{qk}\right|\cos\delta_{pq}+ℜ\left\{h_{(d),kl}\right\}\right)\times \ldots \right.\\ \;\left.\left(\sum_{p=1}^{N}\sum_{q=1}^{N}\left|h_{ip}\right|\left|g_{qk}\right|\cos\delta_{pq}+ℜ\left\{h_{(d),ki}\right\}\right)\right]. \end{array}$$

To compute the expected value $E\left[c_{lk}c_{ik}\right]$, it is needed to change the summation indexes as

(A35) $$\begin{array}{c} E\left[c_{lk}c_{ik}\right]=E\left[\left(\sum_{p_{1}=1}^{N}\sum_{q_{1}=1}^{N}\left|h_{lp_{1}}\right|\left|g_{q_{1}k}\right|\cos\delta_{p_{1}q_{1}}+ℜ\left\{h_{(d),kl}\right\}\right)\times \ldots \right.\\ \;\left.\left(\sum_{p_{2}=1}^{N}\sum_{q_{2}=1}^{N}\left|h_{ip_{2}}\right|\left|g_{q_{2}k}\right|\cos\delta_{p_{2}q_{2}}+ℜ\left\{h_{(d),ki}\right\}\right)\right]. \end{array}$$

After applying the product and considering the independent indexes, it follows that

(A36) $$\begin{array}{c} E\left[c_{lk}c_{ik}\right]=E\left[\sum_{p_{1}=1}^{N}\sum_{q_{1}=1}^{N}\sum_{p_{2}=1}^{N}\sum_{q_{2}=1}^{N}\left|h_{lp_{1}}\right|\left|h_{ip_{2}}\right|\left|g_{q_{1}k}\right|\left|g_{q_{2}k}\right|\cos\delta_{p_{1}q_{1}}\cos\delta_{p_{2}q_{2}}\right]+\\ \;E\left[ℜ\left\{h_{(d),ki}\right\}\sum_{p_{1}=1}^{N}\sum_{q_{1}=1}^{N}\left|h_{lp_{1}}\right|\left|g_{q_{1}k}\right|\cos\delta_{p_{1}q_{1}}\right]+E\left[ℜ\left\{h_{(d),kl}\right\}\sum_{p_{2}=1}^{N}\sum_{q_{2}=1}^{N}\left|h_{ip_{2}}\right|\left|g_{q_{2}k}\right|\cos\delta_{p_{2}q_{2}}\right]+\\ E\left[ℜ\left\{h_{(d),kl}\right\}ℜ\left\{h_{(d),ki}\right\}\right], \end{array}$$

by expanding the previous equations, then

(A37) $$\begin{array}{c} E\left[c_{lk}c_{ik}\right]=\sum_{p_{1}=1}^{N}\sum_{q_{1}=1}^{N}\sum_{p_{2}=1}^{N}\sum_{q_{2}=1}^{N}E\left[\left|h_{lp_{1}}\right|\left|h_{ip_{2}}\right|\right]E\left[\left|g_{q_{1}k}\right|\left|g_{q_{2}k}\right|\right]E\left[\cos\delta_{p_{1}q_{1}}\cos\delta_{p_{2}q_{2}}\right]+\\ E\left[ℜ\{h_{(d),ki}\}\right]\sum_{p_{1}=1}^{N}\sum_{q_{1}=1}^{N}E\left[\left|h_{lp_{1}}\right|\right]E\left[\left|g_{q_{1}k}\right|\right]E\left[\cos\delta_{p_{1}q_{1}}\right]+\\ E\left[ℜ\{h_{(d),kl}\}\right]\sum_{p_{2}=1}^{N}\sum_{q_{2}=1}^{N}E\left[\left|h_{ip_{2}}\right|\right]E\left[\left|g_{q_{2}k}\right|\right]E\left[\cos\delta_{p_{2}q_{2}}\right]+\\ E\left[ℜ\left\{h_{(d),kl}\right\}ℜ\left\{h_{(d),ki}\right\}\right]. \end{array}$$

Assuming that l≠i

(A38) $$\begin{array}{c} E\left[c_{lk}c_{ik}\right]=2N^{2}\times \mu_{ℜ\{h_{\left(d\right)}\}}\left(\mu_{h}\mu_{q}\alpha_{1}\right)+\mu_{ℜ\{h_{\left(d\right)}\}}^{2}+\\ \;N^{2}\left(N-1\right)\left(E\left[{\left|h_{lp_{1}}\right|}^{2}\right]{\left(E\left[\left|g_{q_{1}k}\right|\right]\right)}^{2}{\left(E\left[\left(\cos\delta_{p_{1}q_{1}}\right)\right]\right)}^{2}\right)+\\ \;N^{2}\left(N-1\right)\left(E\left[{\left|h_{lp_{1}}\right|}^{2}\right]E\left[{\left|g_{q_{1}k}\right|}^{2}\right]{\left(E\left[\left(\cos\delta_{p_{1}q_{1}}\right)\right]\right)}^{2}\right)+\\ \;N^{2}{\left(N-1\right)}^{2}\left(E\left[{\left|h_{lp_{1}}\right|}^{2}\right]{\left(E\left[\left|g_{q_{1}k}\right|\right]\right)}^{2}{\left(E\left[\left(\cos\delta_{p_{1}q_{1}}\right)\right]\right)}^{2}\right)+\\ \;N^{2}\left(E\left[{\left|h_{lp_{1}}\right|}^{2}\right]E\left[{\left|g_{q_{1}k}\right|}^{2}\right]E\left[{\left(\cos\delta_{p_{1}q_{1}}\right)}^{2}\right]\right),\forall l\neq i, \end{array}$$

which can be simplified to

(A39) $$\begin{array}{c} E\left[c_{lk}c_{ik}\right]=\frac{N^{2}}{2}\xi_{h}\xi_{g}\left(1+\alpha_{2}\right)+\\ \;N^{2}\left(N-1\right)\xi_{h}\mu_{q}^{2}\alpha_{1}^{2}+N^{2}\left(N-1\right)\xi_{h}\xi_{g}\alpha_{1}^{2}+N^{2}{\left(N-1\right)}^{2}\xi_{h}\mu_{q}^{2}\alpha_{1}^{2}+\\ \;2N^{2}\times \mu_{ℜ\{h_{\left(d\right)}\}}\left(\mu_{h}\mu_{q}\alpha_{1}\right)+\mu_{ℜ\{h_{\left(d\right)}\}}^{2},\forall l\neq i. \end{array}$$

The term $E\left[s_{lk}s_{ik}\right]$, assuming that l≠i, can be computed as follows:

(A40) $$\begin{array}{c} E\left[s_{lk}s_{ik}\right]=E\left[\left(\sum_{p=1}^{N}\sum_{q=1}^{N}\left|h_{lp}\right|\left|g_{qk}\right|\sin\delta_{pq}+ℑ\left\{h_{(d),kl}\right\}\right)\times \ldots \right.\\ \;\left.\left(\sum_{p=1}^{N}\sum_{q=1}^{N}\left|h_{ip}\right|\left|g_{qk}\right|\sin\delta_{pq}+ℑ\left\{h_{(d),ki}\right\}\right)\right], \end{array}$$

by expanding the previous equation, then

(A41) $$\begin{array}{c} E\left[s_{lk}s_{ik}\right]=E\left[\sum_{p_{1}=1}^{N}\sum_{q_{1}=1}^{N}\sum_{p_{2}=1}^{N}\sum_{q_{2}=1}^{N}\left|h_{lp_{1}}\right|\left|h_{ip_{2}}\right|\left|g_{q_{1}k}\right|\left|g_{q_{2}k}\right|\sin\delta_{p_{1}q_{1}}\sin\delta_{p_{2}q_{2}}\right]+\\ \;E\left[ℑ\left\{h_{(d),ki}\right\}\sum_{p_{1}=1}^{N}\sum_{q_{1}=1}^{N}\left|h_{lp_{1}}\right|\left|g_{q_{1}k}\right|\sin\delta_{p_{1}q_{1}}\right]+E\left[ℑ\left\{h_{(d),kl}\right\}\sum_{p_{2}=1}^{N}\sum_{q_{2}=1}^{N}\left|h_{ip_{2}}\right|\left|g_{q_{2}k}\right|\sin\delta_{p_{2}q_{2}}\right]+\\ E\left[ℑ\left\{h_{(d),kl}\right\}ℑ\left\{h_{(d),ki}\right\}\right], \end{array}$$

and can be rewritten as

(A42) $$\begin{array}{c} E\left[s_{lk}s_{ik}\right]=\sum_{p_{1}=1}^{N}\sum_{q_{1}=1}^{N}\sum_{p_{2}=1}^{N}\sum_{q_{2}=1}^{N}E\left[\left|h_{lp_{1}}\right|\left|h_{ip_{2}}\right|\right]E\left[\left|g_{q_{1}k}\right|\left|g_{q_{2}k}\right|\right]E\left[\sin\delta_{p_{1}q_{1}}\sin\delta_{p_{2}q_{2}}\right]+\\ E\left[ℑ\{h_{(d),ki}\}\right]\sum_{p_{1}=1}^{N}\sum_{q_{1}=1}^{N}E\left[\left|h_{lp_{1}}\right|\right]E\left[\left|g_{q_{1}k}\right|\right]E\left[\sin\delta_{p_{1}q_{1}}\right]+\\ E\left[ℑ\{h_{(d),kl}\}\right]\sum_{p_{2}=1}^{N}\sum_{q_{2}=1}^{N}E\left[\left|h_{ip_{2}}\right|\right]E\left[\left|g_{q_{2}k}\right|\right]E\left[\sin\delta_{p_{2}q_{2}}\right]+\\ E\left[ℑ\left\{h_{(d),kl}\right\}ℑ\left\{h_{(d),ki}\right\}\right], \end{array}$$

by substituting the statistical moments of the random variables, it follows that

(A43) $$\begin{array}{c} E\left[s_{lk}s_{ik}\right]=N^{2}\left(E\left[{\left|h_{lp_{1}}\right|}^{2}\right]E\left[{\left|g_{q_{1}k}\right|}^{2}\right]E\left[{\left(\sin\delta_{p_{1}q_{1}}\right)}^{2}\right]\right)+\\ N^{2}\left(N-1\right)\left(E\left[{\left|h_{lp_{1}}\right|}^{2}\right]{\left(E\left[\left|g_{q_{1}k}\right|\right]\right)}^{2}{\left(E\left[\left(\sin\delta_{p_{1}q_{1}}\right)\right]\right)}^{2}\right)+\\ N^{2}\left(N-1\right)\left(E\left[{\left|h_{lp_{1}}\right|}^{2}\right]E\left[{\left|g_{q_{1}k}\right|}^{2}\right]{\left(E\left[\left(\sin\delta_{p_{1}q_{1}}\right)\right]\right)}^{2}\right)+\\ N^{2}{\left(N-1\right)}^{2}\left(E\left[{\left|h_{lp_{1}}\right|}^{2}\right]{\left(E\left[\left|g_{q_{1}k}\right|\right]\right)}^{2}{\left(E\left[\left(\sin\delta_{p_{1}q_{1}}\right)\right]\right)}^{2}\right)+\\ 2N^{2}\times \mu_{ℑ\{h_{\left(d\right)}\}}\left(\mu_{h}\mu_{q}\beta_{1}\right)+\mu_{ℑ\{h_{\left(d\right)}\}}^{2},\mathrm{for}\;l\neq i, \end{array}$$

that can be simplified to

(A44) $$\begin{array}{c} E\left[s_{lk}s_{ik}\right]=\frac{N^{2}}{2}\xi_{h}\xi_{g}\left(1-\alpha_{2}\right)+N^{2}\left(N-1\right)\xi_{h}\mu_{q}^{2}\beta_{1}^{2}+N^{2}\left(N-1\right)\xi_{h}\xi_{g}\beta_{1}^{2}+N^{2}{\left(N-1\right)}^{2}\xi_{h}\mu_{q}^{2}\beta_{1}^{2}+\\ \;2N^{2}\times \mu_{ℑ\{h_{\left(d\right)}\}}\left(\mu_{h}\mu_{q}\beta_{1}\right)+\mu_{ℑ\{h_{\left(d\right)}\}}^{2},\mathrm{for}l\neq i, \end{array}$$

since $\beta_{1}=0$, therefore

(A45) $$E\left[s_{lk}s_{ik}\right]=\frac{N^{2}}{2}\xi_{h}\xi_{g}\left(1-\alpha_{2}\right)+\mu_{ℑ\{h_{\left(d\right)}\}}^{2},\mathrm{for}\;l\neq i.$$

The covariance $cov\left(c_{lk},c_{ik}\right)$ can be computed as

(A46) $$cov\left(c_{lk},c_{ik}\right)=E\left[c_{lk}c_{ik}\right]-{\left(E\left[c_{lk}\right]\right)}^{2},$$

then

(A47) $$\begin{array}{c} cov\left(c_{lk},c_{ik}\right)=\frac{N^{2}}{2}\xi_{h}\xi_{g}\left(1+\alpha_{2}\right)+N^{2}\left(N-1\right)\xi_{h}\mu_{q}^{2}\alpha_{1}^{2}+N^{2}\left(N-1\right)\xi_{h}\xi_{g}\alpha_{1}^{2}+\\ \;N^{2}{\left(N-1\right)}^{2}\xi_{h}\mu_{q}^{2}\alpha_{1}^{2}+2N^{2}\mu_{ℜ\{h_{\left(d\right)}\}}\left(\mu_{h}\mu_{q}\alpha_{1}\right)+\mu_{ℜ\{h_{\left(d\right)}\}}^{2}-N^{4}\mu_{h}^{2}\mu_{g}^{2}\alpha_{1}^{2}, \end{array}$$

and covariance $cov\left(s_{lk},s_{ik}\right)$ can be calculated as

(A48) $$cov\left(s_{lk},s_{ik}\right)=E\left[s_{lk}s_{ik}\right]=\frac{N^{2}}{2}\xi_{h}\xi_{g}\left(1-\alpha_{2}\right)+\mu_{ℑ\{h_{\left(d\right)}\}}^{2}.$$

The variances $var\left(c_{lk}\right)$ and $var\left(s_{lk}\right)$ can be computed as

(A49) $$var\left(c_{lk}\right)=E\left[c_{lk}^{2}\right]-{\left(E\left[c_{lk}\right]\right)}^{2},$$

that can be rewritten as

(A50) $$\begin{array}{c} var\left(c_{lk}\right)=\frac{N^{2}}{2}\xi_{h}\xi_{g}\left(1+\alpha_{2}\right)+N^{2}\left(N-1\right)\xi_{h}\mu_{q}^{2}\alpha_{1}^{2}+N^{2}\left(N-1\right)\left(\mu_{h}^{2}\xi_{g}\alpha_{1}^{2}\right)+\\ \;N^{2}{\left(N-1\right)}^{2}\left(\mu_{h}^{2}\mu_{q}^{2}\alpha_{1}^{2}\right)+2N^{2}\mu_{ℜ\{h_{\left(d\right)}\}}\mu_{h}\mu_{q}\alpha_{1}+\xi_{ℜ\{h_{\left(d\right)}\}}-N^{4}\mu_{h}^{2}\mu_{g}^{2}\alpha_{1}^{2}, \end{array}$$

since

(A51) $$var\left(s_{lk}\right)=E\left[s_{lk}^{2}\right]-{\left(E\left[s_{lk}\right]\right)}^{2}=E\left[s_{lk}^{2}\right],$$

then

(A52) $$var\left(s_{lk}\right)=\frac{N^{2}}{2}\xi_{h}\xi_{g}\left(1-\alpha_{2}\right)+\xi_{ℑ\{h_{\left(d\right)}\}}.$$

The variance $var\left(C_{k}\right)$ can be calculated by

(A53) $$\begin{array}{c} \sigma_{C_{k}}^{2}=var\left(C_{k}\right)=M\left(M-1\right)\left[\frac{N^{2}}{2}\xi_{h}\xi_{g}\left(1+\alpha_{2}\right)+N^{2}\left(N-1\right)\xi_{h}\mu_{q}^{2}\alpha_{1}^{2}+\right.\\ \;\left.N^{2}\left(N-1\right)\xi_{h}\xi_{g}\alpha_{1}^{2}+N^{2}{\left(N-1\right)}^{2}\xi_{h}\mu_{q}^{2}\alpha_{1}^{2}+2N^{2}\mu_{ℜ\{h_{\left(d\right)}\}}\left(\mu_{h}\mu_{q}\alpha_{1}\right)+\mu_{ℜ\{h_{\left(d\right)}\}}^{2}-N^{4}\mu_{h}^{2}\mu_{g}^{2}\alpha_{1}^{2}\right]+\\ \;M\left[\frac{N^{2}}{2}\xi_{h}\xi_{g}\left(1+\alpha_{2}\right)+N^{2}\left(N-1\right)\xi_{h}\mu_{q}^{2}\alpha_{1}^{2}+N^{2}\left(N-1\right)\left(\mu_{h}^{2}\xi_{g}\alpha_{1}^{2}\right)+\right.\\ \;\left.N^{2}{\left(N-1\right)}^{2}\left(\mu_{h}^{2}\mu_{q}^{2}\alpha_{1}^{2}\right)+2N^{2}\mu_{ℜ\{h_{\left(d\right)}\}}\mu_{h}\mu_{q}\alpha_{1}+\xi_{ℜ\{h_{\left(d\right)}\}}-N^{4}\mu_{h}^{2}\mu_{g}^{2}\alpha_{1}^{2}\right]. \end{array}$$

The variance $var\left(S_{k}\right)$ can be defined as

(A54) $$\begin{array}{c} \sigma_{S_{k}}^{2}=var\left(S_{k}\right)=M\left(M-1\right)\left[\frac{N^{2}}{2}\xi_{h}\xi_{g}\left(1-\alpha_{2}\right)+\mu_{ℑ\{h_{\left(d\right)}\}}^{2}\right]+\\ \;M\left[\frac{N^{2}}{2}\xi_{h}\xi_{g}\left(1-\alpha_{2}\right)+\xi_{ℑ\{h_{\left(d\right)}\}}\right], \end{array}$$

and the correlation $E\left[C_{k}S_{k}\right]$ is

(A55) $$\begin{array}{c} E\left[C_{k}S_{k}\right]=E\left[\left(\sum_{l=1}^{M}{\left(\sum_{p=1}^{N}\sum_{q=1}^{N}\left|h_{lp}\right|\left|g_{qk}\right|\cos\delta_{pq}+ℜ\left\{h_{(d),kl}\right\}\right)}^{2}\right)\times \ldots \right.\\ \;\left.\left(\sum_{l=1}^{M}{\left(\sum_{p=1}^{N}\sum_{q=1}^{N}\left|h_{lp}\right|\left|g_{qk}\right|\sin\delta_{pq}+ℑ\left\{h_{(d),kl}\right\}\right)}^{2}\right]\right). \end{array}$$

The term $E\left[C_{k}S_{k}\right]$ can be calculated as

(A56) $$E\left[C_{k}S_{k}\right]=E\left[\sum_{l_{1}=1}^{M}s_{l_{1}k}^{2}\sum_{l_{2}=1}^{M}c_{l_{2}k}^{2}\right]=\sum_{l=1}^{M}\sum_{l_{2}=1}^{M}E\left[s_{l_{1}k}^{2}c_{l_{2}k}^{2}\right],$$

the term $E\left[s_{l_{1}k}^{2}c_{l_{2}k}^{2}\right]$ can be computed as

(A57) $$\begin{array}{c} E\left[s_{l_{1}k}^{2}c_{l_{2}k}^{2}\right]=E\left[\left(\sum_{p_{1}=1}^{N}\sum_{p_{2}=1}^{N}\sum_{q_{1}=1}^{N}\sum_{q_{2}=1}^{N}\left|h_{l_{1}p_{1}}\right|\left|h_{l_{1}p_{2}}\right|\left|g_{q_{1}k}\right|\left|g_{q_{2}k}\right|\sin\delta_{p_{1}q_{1}}\sin\delta_{p_{2}q_{2}}+\right.\right.\\ \left.\left.{\left(ℑ\{h_{\left(d\right),kl_{1}}\}\right)}^{2}+2ℑ\left\{h_{\left(d\right),kl_{1}}\right\}\sum_{p_{1}=1}^{N}\sum_{q_{1}=1}^{N}\left|h_{l_{1}p_{1}}\right|\left|g_{q_{1}k}\right|\sin\delta_{p_{1}q_{1}}\right)\times \right.\\ \left.\left(\sum_{p_{3}=1}^{N}\sum_{p_{4}=1}^{N}\sum_{q_{3}=1}^{N}\sum_{q_{4}=1}^{N}\left|h_{l_{2}p_{3}}\right|\left|h_{l_{2}p_{4}}\right|\left|g_{q_{3}k}\right|\left|g_{q_{4}k}\right|\cos\delta_{p_{3}q_{3}}\cos\delta_{p_{4}q_{4}}+{\left(ℜ\{h_{\left(d\right),kl_{2}}\}\right)}^{2}+\right.\right.\\ \left.\left.2ℜ\left\{h_{\left(d\right),kl_{2}}\right\}\sum_{p_{3}=1}^{N}\sum_{q_{3}=1}^{N}\left|h_{l_{2}p_{3}}\right|\left|g_{q_{3}k}\right|\cos\delta_{p_{3}q_{3}}\right)\right], \end{array}$$

then, $\mu_{C_{k}S_{k}}$ will be given by (A58).

(A58) $$\begin{array}{c} \mu_{C_{k}S_{k}}=M\left(M-1\right)\left[{\left(N-1\right)}^{4}b_{10}b_{18}+N^{2}b_{14}b_{17}b_{29}\alpha_{1}^{2}+Nb_{14}b_{27}\mu_{g}\chi_{g}b_{28}+Nb_{12}b_{27}\mu_{g}\chi_{g}b_{28}+\right.\\ \left.2b_{12}b_{27}\epsilon_{g}b_{28}+b_{12}\mu_{h}b_{23}\chi_{h}b_{28}+4b_{8}\mu_{h}^{2}\xi_{h}\chi_{g}\mu_{g}b_{28}+2N^{2}\left(N-1\right)\mu_{h}^{2}\xi_{h}\epsilon_{g}b_{28}+b_{12}b_{17}b_{28}+\right.\\ \left.b_{8}b_{23}\xi_{h}^{2}b_{28}+N^{2}\left(N-1\right)b_{23}\xi_{h}^{2}b_{28}+N^{2}\left(N-1\right)\mu_{g}\xi_{h}^{2}\chi_{g}b_{28}+N^{2}\xi_{h}^{2}\epsilon_{g}b_{28}+\right.\\ \left.\left(N-1\right)N^{2}\xi_{ℜ\{h_{\left(d\right)}\}}b_{25}b_{29}+N^{2}\xi_{ℜ\{h_{\left(d\right)}\}}\xi_{h}\xi_{g}b_{29}+N^{2}\left(\left(N-1\right)\xi_{ℑ\{h_{\left(d\right)}\}}b_{25}\alpha_{1}^{2}+\xi_{h}\xi_{g}b_{1}\right)+\right.\\ \left.\xi_{ℜ\{h_{\left(d\right)}\}}+b_{8}\xi_{ℑ\{h_{\left(d\right)}\}}b_{24}+N^{2}\left(N-1\right)\xi_{ℑ\{h_{\left(d\right)}\}}\mu_{h}^{2}\xi_{g}\alpha_{1}^{2}+2\xi_{ℜ\{h_{\left(d\right)}\}}\right]+M\left[{\left(N-1\right)}^{4}b_{10}b_{18}+\right.\\ \left.N^{2}b_{14}b_{17}b_{29}\alpha_{1}^{2}+Nb_{14}b_{27}\mu_{g}\chi_{g}b_{28}+N^{2}b_{14}b_{27}\mu_{g}\chi_{g}b_{28}+2b_{12}b_{27}\epsilon_{g}b_{28}+b_{12}b_{27}\mu_{g}\chi_{g}b_{28}+\right.\\ \left.b_{12}\mu_{h}b_{23}\chi_{h}b_{28}+2N^{2}\left(N-1\right)\mu_{h}\chi_{h}\epsilon_{g}b_{28}+b_{12}\mu_{g}^{2}\chi_{h}\mu_{h}\xi_{g}b_{28}+4b_{8}\mu_{g}\chi_{h}\mu_{h}\chi_{g}b_{28}+b_{8}b_{23}\epsilon_{h}b_{28}+\right.\\ \left.N^{2}\left(N-1\right)b_{23}\epsilon_{h}b_{28}+N^{2}\left(N-1\right)\mu_{g}\epsilon_{h}\chi_{g}b_{28}+N^{2}\epsilon_{h}\epsilon_{g}b_{28}+\right.\\ \left.\left(N-1\right)N^{2}\xi_{ℜ\{h_{\left(d\right)}\}}\xi_{h}\mu_{g}^{2}b_{29}+\xi_{ℜ\{h_{\left(d\right)}\}}N^{2}\xi_{h}\xi_{g}b_{29}+N^{2}\xi_{ℑ\{h_{\left(d\right)}\}}\left(\left(N-1\right)b_{25}\alpha_{1}^{2}+\xi_{h}\xi_{g}b_{1}\right)+\right.\\ \left.\xi_{ℜ\{h_{\left(d\right)}\}}+b_{8}\xi_{ℑ\{h_{\left(d\right)}\}}b_{24}+N^{2}\left(N-1\right)\xi_{ℑ\{h_{\left(d\right)}\}}\mu_{h}^{2}\xi_{g}\alpha_{1}^{2}+2\xi_{ℜ\{h_{\left(d\right)}\}}\right]. \end{array}$$

The terms $b_{i}$ can be computed as $b_{1}=\frac{1}{2}\left(1+\alpha_{2}\right)$, $b_{2}=\frac{1}{8}\left(3+4\alpha_{2}+\alpha_{4}\right)$, $b_{3}=\alpha_{1}^{2}\left(1+\alpha_{2}\right)$, $b_{4}=\frac{1}{4}\left(3\alpha_{1}+\alpha_{3}\right)\alpha_{1}$, $b_{5}=b_{1}\alpha_{1}^{2}$, $b_{6}=N^{2}b_{5}$, $b_{7}=\left(N^{2}-1\right)N^{2}\alpha_{1}^{4}$, $b_{8}={\left(N-1\right)}^{2}N^{2}$, $b_{9}=N{\left(N-1\right)}^{2}$, $b_{1}0=\left(N-1\right)N^{2}\alpha_{1}^{4}$, $b_{1}1=N{\left(N-1\right)}^{3}$, $b_{1}2=N^{2}{\left(N-1\right)}^{3}$, $b_{1}3={\left(N-1\right)}^{3}$, $b_{1}4={\left(N-1\right)}^{4}$, $b_{15}=\mu_{g}^{4}b_{1}\alpha_{1}^{2}$, $b_{16}=\mu_{h}\chi_{h}\mu_{g}^{4}$, $b_{17}=\mu_{h}^{2}\xi_{h}\xi_{g}\mu_{g}^{2}$, $b_{18}=\mu_{h}^{2}\xi_{h}\mu_{g}^{4}$, $b_{19}=\mu_{h}^{4}\xi_{g}\mu_{g}^{2}$, $b_{20}=\mu_{h}^{4}\mu_{g}^{4}b_{10}$, $b_{21}=\mu_{h}\chi_{h}\xi_{g}$, $b_{22}=\mu_{h}\chi_{h}\xi_{g}^{2}$, $b_{23}=\xi_{g}\mu_{g}^{2}$, $b_{24}=\mu_{g}^{2}\mu_{h}^{2}\alpha_{1}^{2}$, $b_{25}=\xi_{h}\mu_{g}^{2}$, $b_{26}=\mu_{g}^{2}b_{1}\alpha_{1}^{2}$, $b_{27}=\xi_{h}\mu_{h}^{2}$, $b_{28}=\alpha_{1}^{2}\frac{1-\alpha_{2}}{2}$, $b_{29}=\frac{1-\alpha_{2}}{2}$.

Given $\mu_{C_{k}S_{k}}$, it follows that

(A59) $$cov\left(C_{k},S_{k}\right)=\mu_{C_{k}S_{k}}-\mu_{C_{k}}\mu_{S_{k}},$$

therefore, the variance will be

(A60) $$\sigma_{Z_{k}}^{2}={\left(\frac{P}{K}\right)}^{2}\left[\sigma_{C_{k}}^{2}+\sigma_{S_{k}}^{2}+2\left(\mu_{C_{k}S_{k}}-\mu_{C_{k}}\mu_{S_{k}}\right)\right].$$

## Appendix B. Statistical Parameters of *F_k_*

### Appendix B.1. Expected Value of F_k_

Since

(A61) $$F_{k}=\sigma_{\eta }^{2}+\sum_{i=0,i\neq k}^{K}Z_{i},$$

then, the expected value of $F_{k}$ can be calculated as

(A62) $$E\left[F_{k}\right]=\sigma_{\eta }^{2}+E\left[\sum_{i=0,i\neq k}^{K}Z_{i}\right]=\sigma_{\eta }^{2}+\left(K-1\right)E\left[Z_{i}\right],$$

since the expected value of $Z_{k}$ was previously computed, therefore

(A63) $$\begin{array}{c} E\left[F_{k}\right]=\sigma_{\eta }^{2}+\left(K-1\right)\frac{PM}{K}\left[\frac{N^{2}}{2}\xi_{h}\xi_{g}\left(1+\alpha_{2}\right)+N^{2}\left(N-1\right)\xi_{h}\mu_{q}^{2}\alpha_{1}^{2}+N^{2}\left(N-1\right)\left(\mu_{h}^{2}\xi_{g}\alpha_{1}^{2}\right)+\right.\\ \;\left.N^{2}{\left(N-1\right)}^{2}\left(\mu_{h}^{2}\mu_{q}^{2}\alpha_{1}^{2}\right)+2N^{2}\mu_{ℜ\{h_{\left(d\right)}\}}\mu_{h}\mu_{q}\alpha_{1}+\xi_{ℜ\{h_{\left(d\right)}\}}+\frac{N^{2}}{2}\xi_{h}\xi_{g}\left(1-\alpha_{2}\right)+\xi_{ℑ\{h_{\left(d\right)}\}}\right]. \end{array}$$

### Appendix B.2. Variance of F_k_

The variance $var\left(F_{k}\right)$ can be defined in terms of the statistics of $Z_{i}$ as

(A64) $$\sigma_{F_{k}}^{2}=var\left(F_{k}\right)=var\left(\sigma_{\eta }^{2}+\sum_{i=0,i\neq k}^{K}Z_{i}\right)=var\left(\sum_{i=0,i\neq k}^{K}Z_{i}\right),$$

since the terms are independent and equally likely, then

(A65) $$var\left(F_{k}\right)=K\left(K-1\right)cov\left(Z_{i},Z_{t}\right)+Kvar\left(Z_{i}\right),$$

considering that

(A66) $$cov\left(Z_{i},Z_{t}\right)=E\left[Z_{i}Z_{t}\right]-{\left(E[Z_{i}]\right)}^{2}$$

where

(A67) $$E\left[Z_{i}Z_{t}\right]={\left(\frac{P}{K}\right)}^{2}E\left[R_{i}R_{t}\right],$$

that can be rewritten as

(A68) $$E\left[Z_{i}Z_{t}\right]={\left(\frac{P}{K}\right)}^{2}E\left[\left(C_{i}+S_{i}\right)\left(C_{t}+S_{t}\right)\right],$$

by expanding the product, it follows that

(A69) $$E\left[Z_{i}Z_{t}\right]={\left(\frac{P}{K}\right)}^{2}E\left[(C_{i}C_{t}+S_{i}S_{t}+C_{i}S_{t}+S_{i}C_{t})\right],$$

since $E\left[C_{i}S_{t}\right]=E\left[S_{i}C_{t}\right]$, then

(A70) $$E\left[(C_{i}C_{t}+S_{i}S_{t}+C_{i}S_{t}+S_{i}C_{t})\right]=E\left[C_{i}C_{t}\right]+E\left[S_{i}S_{t}\right]+2E\left[S_{i}C_{t}\right].$$

For i≠t

(A71) $$E\left[C_{i}C_{t}\right]=E\left[\sum_{l_{1}=1}^{M}c_{l_{1}i}^{2}\sum_{l_{2}=1}^{M}c_{l_{2}t}^{2}\right]=\sum_{l_{1}=1}^{M}\sum_{l_{2}=1}^{M}E\left[c_{l_{1}i}^{2}c_{l_{2}t}^{2}\right],$$

by expanding the terms, it follows that

(A72) $$\begin{array}{c} E\left[c_{l_{1}i}^{2}c_{l_{2}t}^{2}\right]=E\left[\left(\sum_{p_{1}=1}^{N}\sum_{p_{2}=1}^{N}\sum_{q_{1}=1}^{N}\sum_{q_{2}=1}^{N}\left|h_{l_{1}p_{1}}\right|\left|h_{l_{1}p_{2}}\right|\left|g_{q_{1}i}\right|\left|g_{q_{2}i}\right|\cos\delta_{p_{1}q_{1}}\cos\delta_{p_{2}q_{2}}+\right.\right.\\ \left.\left.{\left(ℜ\{h_{\left(d\right),il_{1}}\}\right)}^{2}+2ℜ\left\{h_{\left(d\right),il_{1}}\right\}\sum_{p_{1}=1}^{N}\sum_{q_{1}=1}^{N}\left|h_{l_{1}p_{1}}\right|\left|g_{q_{1}i}\right|\cos\delta_{p_{1}q_{1}}\right)\times \right.\\ \left.\left(\sum_{p_{3}=1}^{N}\sum_{p_{4}=1}^{N}\sum_{q_{3}=1}^{N}\sum_{q_{4}=1}^{N}\left|h_{l_{2}p_{3}}\right|\left|h_{l_{2}p_{4}}\right|\left|g_{q_{3}t}\right|\left|g_{q_{4}t}\right|\cos\delta_{p_{3}q_{3}}\cos\delta_{p_{4}q_{4}}+{\left(ℜ\{h_{\left(d\right),tl_{2}}\}\right)}^{2}+\right.\right.\\ \left.\left.2ℜ\left\{h_{\left(d\right),tl_{2}}\right\}\sum_{p_{3}=1}^{N}\sum_{q_{3}=1}^{N}\left|h_{l_{2}p_{3}}\right|\left|g_{q_{3}t}\right|\cos\delta_{p_{3}q_{3}}\right)\right]. \end{array}$$

By expanding the terms of $E[c_{l_{1}i}^{2}c_{l_{2}t}^{2}]$, the expected value can be rewritten as (A73).

(A73) $$\begin{array}{c} E\left[c_{l_{1}i}^{2}c_{l_{2}t}^{2}\right]=\sum_{p_{1}=1}^{N}\sum_{p_{2}=1}^{N}\sum_{p_{3}=1}^{N}\sum_{p_{4}=1}^{N}\sum_{q_{1}=1}^{N}\sum_{q_{2}=1}^{N}\sum_{q_{3}=1}^{N}\sum_{q_{4}=1}^{N}E\left[\left|h_{l_{1}p_{1}}\right|\left|h_{l_{1}p_{2}}\right|\left|h_{l_{2}p_{3}}\right|\left|h_{l_{2}p_{4}}\right|\right]\times \\ E\left[\left|g_{q_{1}i}\right|\left|g_{q_{2}i}\right|\left|g_{q_{3}t}\right|\left|g_{q_{4}t}\right|\right]E\left[\cos\delta_{p_{1}q_{1}}\cos\delta_{p_{2}q_{2}}\cos\delta_{p_{3}q_{3}}\cos\delta_{p_{4}q_{4}}\right]+\\ \xi_{ℜ\{h_{\left(d\right)}\}}\sum_{p_{1}=1}^{N}\sum_{p_{2}=1}^{N}\sum_{q_{1}=1}^{N}\sum_{q_{2}=1}^{N}E\left[\left|h_{l_{1}p_{1}}\right|\left|h_{l_{1}p_{2}}\right|\right]E\left[\left|g_{q_{1}i}\right|\left|g_{q_{2}i}\right|\right]E\left[\cos\delta_{p_{1}q_{1}}\cos\delta_{p_{2}q_{2}}\right]+\\ \xi_{ℜ\{h_{\left(d\right)}\}}\sum_{p_{3}=1}^{N}\sum_{p_{4}=1}^{N}\sum_{q_{3}=1}^{N}\sum_{q_{4}=1}^{N}E\left[\left|h_{l_{2}p_{3}}\right|\left|h_{l_{2}p_{4}}\right|\left|g_{q_{3}t}\right|\left|g_{q_{4}t}\right|\cos\delta_{p_{3}q_{3}}\cos\delta_{p_{4}q_{4}}\right]+\\ E\left[{\left(ℜ\{h_{\left(d\right),il_{1}}\}\right)}^{2}{\left(ℜ\{h_{\left(d\right),tl_{2}}\}\right)}^{2}\right]+\\ 2E\left[{\left(ℜ\{h_{\left(d\right),il_{1}}\}\right)}^{2}ℜ\left\{h_{\left(d\right),tl_{2}}\right\}\right]\sum_{p_{3}=1}^{N}\sum_{q_{3}=1}^{N}E\left[\left|h_{l_{2}p_{3}}\right|\right]E\left[\left|g_{q_{3}t}\right|\right]\alpha_{1}+\\ 2E\left[{\left(ℜ\{h_{\left(d\right),tl_{2}}\}\right)}^{2}ℜ\left\{h_{\left(d\right),il_{1}}\right\}\right]\sum_{p_{1}=1}^{N}\sum_{q_{1}=1}^{N}E\left[\left|h_{l_{1}p_{1}}\right|\right]E\left[\left|g_{q_{1}i}\right|\right]\alpha_{1}+\\ 4E\left[ℜ\left\{h_{\left(d\right),il_{1}}\right\}ℜ\left\{h_{\left(d\right),tl_{2}}\right\}\right]\sum_{p_{1}=1}^{N}\sum_{q_{1}=1}^{N}\sum_{p_{3}=1}^{N}\sum_{q_{3}=1}^{N}E\left[\left|h_{l_{1}p_{1}}\right|\left|h_{l_{2}p_{3}}\right|\right]E\left[\left|g_{q_{1}i}\right|\left|g_{q_{3}t}\right|\right]E\left[\cos\delta_{p_{1}q_{1}}\cos\delta_{p_{3}q_{3}}\right] \end{array}$$

Through the recursive expansion of the expression and analyzing the varying scenarios where the variables might be interdependent or independent, based on their index values, it can be inferred that, for $l_{1}\neq l_{2}$, the expected value $E[c_{l_{1}i}^{2}c_{l_{2}t}^{2}]$ will be given by (A74). For $l_{1}=l_{2}$, it can be calculated by (A75).

(A74) $$\begin{array}{c} E\left[c_{l_{1}i}^{2}c_{l_{2}t}^{2}\right]=b_{8}\xi_{ℜ\{h_{\left(d\right)}\}}b_{24}+Nb_{9}\xi_{ℜ\{h_{\left(d\right)}\}}\left(b_{24}+b_{25}\alpha_{1}^{2}+2\mu_{h}^{2}\xi_{g}\right)+b_{14}N^{2}b_{17}\alpha_{4}+b_{14}b_{20}\\ \xi {ℜh\left(d\right)}^{2}N^{2}\xi_{ℜ\{h_{\left(d\right)}\}}\left(2\xi_{g}\xi_{h}b_{1}+b_{25}\alpha_{1}^{2}\right){\left(N-1\right)}^{5}b_{20}+b_{14}b_{18}b_{10}+b_{14}b_{18}b_{10}+b_{13}b_{16}b_{10}+\\ b_{14}b_{18}b_{10}+b_{14}b_{19}b_{10}+b_{14}N^{2}b_{17}b_{5}+b_{13}b_{19}b_{10}+b_{13}b_{17}b_{10}+b_{12}b_{21}\mu_{g}^{2}b_{5}+b_{13}b_{18}b_{10}+\\ b_{14}N^{2}b_{18}b_{5}+{\left(N-1\right)}^{2}b_{17}b_{10}+b_{12}b_{16}b_{5}+b_{13}b_{27}\mu_{g}^{4}b_{10}+b_{13}b_{19}b_{10}+b_{12}b_{17}b_{5}+b_{12}b_{17}b_{5}+\\ b_{8}b_{21}\mu_{g}^{2}b_{4}+{\left(N-1\right)}^{2}b_{17}b_{10}+b_{14}b_{20}+b_{13}b_{18}b_{10}+b_{13}b_{10}b_{18}+{\left(N-1\right)}^{2}b_{16}b_{10}+b_{14}N^{2}b_{18}b_{5}+\\ b_{12}b_{17}b_{1}+{\left(N-1\right)}^{2}b_{17}b_{10}+b_{8}b_{21}\mu_{g}^{2}b_{5}+b_{12}b_{17}b_{5}+b_{13}b_{19}b_{10}+b_{12}b_{17}b_{5}+\\ b_{8}\mu_{h}\chi_{h}b_{23}b_{5}+b_{12}b_{17}b_{5}+{\left(N-1\right)}^{2}\mu_{h}^{4}\xi_{g}^{2}b_{10}+b_{8}b_{27}\xi_{g}^{2}b_{5}+\left(N-1\right)N^{2}b_{22}b_{4}+b_{8}b_{27}\xi_{g}^{2}b_{5}+\\ b_{8}b_{27}\xi_{g}^{2}b_{5}+{\left(N-1\right)}^{4}N\mu_{h}\chi_{h}\mu_{g}^{4}\alpha_{1}^{4}+b_{11}\mu_{h}\chi_{h}b_{23}b_{1}\alpha_{1}^{2}+b_{1}1\mu_{h}\chi_{h}\mu_{g}^{4}\alpha_{1}^{4}+b_{9}b_{21}b_{26}+b_{1}1b_{21}b_{26}+\\ b_{9}b_{21}\mu_{g}^{2}b_{4}+b_{9}b_{21}b_{26}+N\left(N-1\right)b_{22}b_{4}+{\left(N-1\right)}^{4}Nb_{16}\alpha_{1}^{4}+b_{11}\mu_{h}\chi_{h}b_{15}+b_{11}\mu_{h}\chi_{h}b_{15}+\\ b_{9}b_{21}\mu_{g}^{2}b_{4}+b_{11}b_{21}\mu_{g}^{2}\alpha_{1}^{4}+2b_{9}b_{21}b_{26}+\left(N-1\right)Nb_{22}b_{4}+b_{11}\epsilon_{h}\mu_{g}^{4}\alpha_{1}^{4}+2b_{9}\epsilon_{h}b_{15}+\\ N\left(N-1\right)\epsilon_{h}b_{23}b_{4}+b_{9}\epsilon_{h}b_{23}b_{1}\alpha_{1}^{2}+2N\left(N-1\right)\epsilon_{h}b_{23}b_{4}+N\epsilon_{h}\xi_{g}^{2}b_{2},l_{1}=l_{2} \end{array}$$

(A75) $$\begin{array}{c} E\left[c_{l_{1}i}^{2}c_{l_{2}t}^{2}\right]=b_{8}\xi_{ℜ\{h_{\left(d\right)}\}}b_{24}+Nb_{9}\xi_{ℜ\{h_{\left(d\right)}\}}b_{24}+\left(N-1\right)N^{2}\xi_{ℜ\{h_{\left(d\right)}\}}\left(b_{25}\alpha_{1}^{2}+2\mu_{h}^{2}\xi_{g}\right)+\\ N^{2}\xi_{ℜ\{h_{\left(d\right)}\}}\left(2\xi_{g}\xi_{h}b_{1}+b_{25}\alpha_{1}^{2}\right)+\xi {ℜh\left(d\right)}^{2}+Nb_{14}\mu_{g}^{4}\mu_{h}^{4}\left(\left(N^{2}-1\right)N^{2}b_{1}^{2}+N^{2}b_{2}\right)+\\ b_{11}\mu_{g}^{4}b_{27}\left(b_{8}\alpha_{1}^{3}+N^{2}\left(N-1\right)b_{3}+N^{2}b_{4}\right)+b_{11}b_{27}\mu_{g}^{4}\left(N^{2}\frac{1}{2}\left(\left(N^{2}-1\right)b_{3}+2b_{4}\right)\right)+\\ b_{9}\xi_{h}^{2}\mu_{g}^{4}\left(N^{2}\alpha_{1}^{2}\left[{\left(N-1\right)}^{2}\alpha_{1}^{2}+\left(N-1\right)\alpha_{1}^{2}+b_{1}\right]\right)+b_{9}b_{17}\left(b_{8}b_{5}+2\left(N-1\right)b_{6}+N^{2}b_{4}\right)+\\ b_{9}b_{17}\left(b_{8}\alpha_{1}^{4}+2b_{10}+b_{6}\right)+b_{11}\mu_{h}^{4}\xi_{g}\mu_{g}^{2}\left(b_{6}\left(N^{2}-1\right)+N^{2}b_{4}\right)+b_{11}\xi_{g}\mu_{g}^{2}\mu_{h}^{4}\left(\left(N^{2}-1\right)b_{6}+N^{2}b_{4}\right)+\\ b_{9}b_{17}\left(b_{7}+b_{6}\right)+b_{9}b_{17}\left(b_{8}b_{5}+2\left(N-1\right)b_{6}+N^{2}b_{4}\right)+N\left(N-1\right)\xi_{g}\xi_{h}^{2}\mu_{g}^{2}\left(b_{7}+b_{6}\right)+\\ N\left(N-1\right)\xi_{h}^{2}\mu_{g}^{2}\xi_{g}\left(b_{7}+b_{6}\right)+b_{9}\mu_{h}^{4}\xi_{g}^{2}\left(b_{7}+b_{6}\right)+N\left(N-1\right)\xi_{g}^{2}b_{27}\left(b_{8}\alpha_{1}^{4}+2b_{10}+b_{6}\right)+\\ N\left(N-1\right)b_{27}\xi_{g}^{2}\left(b_{7}+b_{6}\right)+N\xi_{g}^{2}\xi_{h}^{2}\left(b_{7}+b_{6}\right),\forall l_{1}\neq l_{2}. \end{array}$$

Therefore, $E\left[C_{i}C_{t}\right]$ can be calculated by (A76).

(A76) $$\begin{array}{c} \mu_{C_{i}C_{t}}=E\left[C_{i}C_{t}\right]=M\left(M-1\right)\left[Nb_{9}\xi_{ℜ\{h_{\left(d\right)}\}}b_{24}+\left(N-1\right)N^{2}\xi_{ℜ\{h_{\left(d\right)}\}}\left(b_{25}\alpha_{1}^{2}+2\mu_{h}^{2}\xi_{g}\right)+\right.\\ \left.b_{8}\xi_{ℜ\{h_{\left(d\right)}\}}b_{24}+N^{2}\xi_{ℜ\{h_{\left(d\right)}\}}\left(2\xi_{g}\xi_{h}b_{1}+b_{25}\alpha_{1}^{2}\right)+\xi {ℜh\left(d\right)}^{2}+\right.\\ \left.Nb_{14}\mu_{g}^{4}\mu_{h}^{4}\left(\left(N^{2}-1\right)N^{2}b_{1}^{2}+N^{2}b_{2}\right)+b_{11}\mu_{g}^{4}b_{27}\left(b_{8}\alpha_{1}^{3}+N^{2}\left(N-1\right)b_{3}+N^{2}b_{4}\right)+\right.\\ \left.b_{11}b_{27}\mu_{g}^{4}\left(N^{2}\frac{1}{2}\left(\left(N^{2}-1\right)b_{3}+2b_{4}\right)\right)+b_{9}\xi_{h}^{2}\mu_{g}^{4}\left(N^{2}\alpha_{1}^{2}\left[{\left(N-1\right)}^{2}\alpha_{1}^{2}+\left(N-1\right)\alpha_{1}^{2}+b_{1}\right]\right)+\right.\\ \left.b_{9}b_{17}\left(b_{8}b_{5}+2\left(N-1\right)b_{6}+N^{2}b_{4}\right)+b_{9}b_{17}\left(b_{8}\alpha_{1}^{4}+2b_{10}+b_{6}\right)+\right.\\ \left.b_{11}\mu_{h}^{4}\xi_{g}\mu_{g}^{2}\left(b_{6}\left(N^{2}-1\right)+N^{2}b_{4}\right)+b_{11}\xi_{g}\mu_{g}^{2}\mu_{h}^{4}\left(\left(N^{2}-1\right)b_{6}+N^{2}b_{4}\right)+\right.\\ \left.b_{9}b_{17}\left(b_{7}+b_{6}\right)+b_{9}b_{17}\left(b_{8}b_{5}+2\left(N-1\right)b_{6}+N^{2}b_{4}\right)+\right.\\ \left.N\left(N-1\right)\xi_{g}\xi_{h}^{2}\mu_{g}^{2}\left(b_{7}+b_{6}\right)+N\left(N-1\right)\xi_{h}^{2}\mu_{g}^{2}\xi_{g}\left(b_{7}+b_{6}\right)+\right.\\ \left.b_{9}\mu_{h}^{4}\xi_{g}^{2}\left(b_{7}+b_{6}\right)+N\left(N-1\right)\xi_{g}^{2}b_{27}\left(b_{8}\alpha_{1}^{4}+2b_{10}+b_{6}\right)+\right.\\ \left.N\left(N-1\right)b_{27}\xi_{g}^{2}\left(b_{7}+b_{6}\right)+N\xi_{g}^{2}\xi_{h}^{2}\left(b_{7}+b_{6}\right)\right]+M\left[b_{8}\xi_{ℜ\{h_{\left(d\right)}\}}b_{24}+\right.\\ \left.Nb_{9}\xi_{ℜ\{h_{\left(d\right)}\}}\left(b_{24}+b_{25}\alpha_{1}^{2}+2\mu_{h}^{2}\xi_{g}\right)+N^{2}\xi_{ℜ\{h_{\left(d\right)}\}}\left(2\xi_{g}\xi_{h}b_{1}+b_{25}\alpha_{1}^{2}\right)+\xi {ℜh\left(d\right)}^{2}+\right.\\ \left.{\left(N-1\right)}^{5}b_{20}+b_{14}b_{18}b_{10}+b_{14}b_{18}b_{10}+b_{13}b_{16}b_{10}+\right.\\ \left.b_{14}b_{18}b_{10}+b_{14}b_{19}b_{10}+b_{14}N^{2}b_{17}b_{5}+b_{14}N^{2}b_{17}\alpha_{4}+b_{14}b_{20}\right.\\ \left.b_{13}b_{19}b_{10}+b_{13}b_{17}b_{10}+b_{12}b_{21}\mu_{g}^{2}b_{5}+b_{13}b_{18}b_{10}+b_{14}N^{2}b_{18}b_{5}+\right.\\ \left.{\left(N-1\right)}^{2}b_{17}b_{10}+b_{12}b_{16}b_{5}+b_{13}b_{27}\mu_{g}^{4}b_{10}+b_{13}b_{19}b_{10}+\right.\\ \left.b_{12}b_{17}b_{5}+b_{12}b_{17}b_{5}+b_{8}b_{21}\mu_{g}^{2}b_{4}+{\left(N-1\right)}^{2}b_{17}b_{10}+b_{14}b_{20}+\right.\\ \left.b_{13}b_{18}b_{10}+b_{13}b_{10}b_{18}+{\left(N-1\right)}^{2}b_{16}b_{10}+b_{14}N^{2}b_{18}b_{5}+\right.\\ \left.b_{12}b_{17}b_{1}+{\left(N-1\right)}^{2}b_{17}b_{10}+b_{8}b_{21}\mu_{g}^{2}b_{5}+b_{12}b_{17}b_{5}+b_{13}b_{19}b_{10}+\right.\\ \left.b_{12}b_{17}b_{5}+b_{8}\mu_{h}\chi_{h}b_{23}b_{5}+b_{12}b_{17}b_{5}+{\left(N-1\right)}^{2}\mu_{h}^{4}\xi_{g}^{2}b_{10}+b_{8}b_{27}\xi_{g}^{2}b_{5}+\right.\\ \left.\left(N-1\right)N^{2}b_{22}b_{4}+b_{8}b_{27}\xi_{g}^{2}b_{5}+b_{8}b_{27}\xi_{g}^{2}b_{5}+{\left(N-1\right)}^{4}N\mu_{h}\chi_{h}\mu_{g}^{4}\alpha_{1}^{4}+b_{11}\mu_{h}\chi_{h}b_{23}b_{1}\alpha_{1}^{2}+\right.\\ \left.b_{1}1\mu_{h}\chi_{h}\mu_{g}^{4}\alpha_{1}^{4}+b_{9}b_{21}b_{26}+b_{1}1b_{21}b_{26}+b_{9}b_{21}\mu_{g}^{2}b_{4}+b_{9}b_{21}b_{26}+\right.\\ \left.\left(N-1\right)Nb_{22}b_{4}+{\left(N-1\right)}^{4}Nb_{16}\alpha_{1}^{4}+b_{11}\mu_{h}\chi_{h}b_{15}+\right.\\ \left.b_{11}\mu_{h}\chi_{h}b_{15}+b_{9}b_{21}\mu_{g}^{2}b_{4}+b_{11}b_{21}\mu_{g}^{2}\alpha_{1}^{4}+2b_{9}b_{21}b_{26}+\left(N-1\right)Nb_{22}b_{4}+b_{11}\epsilon_{h}\mu_{g}^{4}\alpha_{1}^{4}+\right.\\ \left.b_{9}\epsilon_{h}b_{15}+b_{9}\epsilon_{h}b_{15}+N\left(N-1\right)\epsilon_{h}b_{23}b_{4}+b_{9}\epsilon_{h}b_{23}b_{1}\alpha_{1}^{2}+2N\left(N-1\right)\epsilon_{h}b_{23}b_{4}+N\epsilon_{h}\xi_{g}^{2}b_{2}\right] \end{array}$$

The expected value $E\left[S_{i}S_{t}\right]$, for i≠t

(A77) $$E\left[S_{i}S_{t}\right]=E\left[\sum_{l_{1}=1}^{M}s_{l_{1}i}^{2}\sum_{l_{2}=1}^{M}s_{l_{2}t}^{2}\right]=\sum_{l_{1}=1}^{M}\sum_{l_{2}=1}^{M}E\left[s_{l_{1}i}^{2}s_{l_{2}t}^{2}\right],$$

where the expected value $E\left[s_{l_{1}i}^{2}s_{l_{2}t}^{2}\right]$ can be calculated as

(A78) $$\begin{array}{c} E\left[s_{l_{1}i}^{2}s_{l_{2}t}^{2}\right]=E\left[\left(\sum_{p_{1}=1}^{N}\sum_{p_{2}=1}^{N}\sum_{q_{1}=1}^{N}\sum_{q_{2}=1}^{N}\left|h_{l_{1}p_{1}}\right|\left|h_{l_{1}p_{2}}\right|\left|g_{q_{1}i}\right|\left|g_{q_{2}i}\right|\sin\delta_{p_{1}q_{1}}\sin\delta_{p_{2}q_{2}}+\right.\right.\\ \left.\left.{\left(ℑ\{h_{\left(d\right),il_{1}}\}\right)}^{2}+2ℑ\left\{h_{\left(d\right),il_{1}}\right\}\sum_{p_{1}=1}^{N}\sum_{q_{1}=1}^{N}\left|h_{l_{1}p_{1}}\right|\left|g_{q_{1}i}\right|\sin\delta_{p_{1}q_{1}}\right)\times \right.\\ \left.\left(\sum_{p_{3}=1}^{N}\sum_{p_{4}=1}^{N}\sum_{q_{3}=1}^{N}\sum_{q_{4}=1}^{N}\left|h_{l_{2}p_{3}}\right|\left|h_{l_{2}p_{4}}\right|\left|g_{q_{3}t}\right|\left|g_{q_{4}t}\right|\sin\delta_{p_{3}q_{3}}\sin\delta_{p_{4}q_{4}}+\right.\right.\\ \left.\left.{\left(ℑ\{h_{\left(d\right),tl_{2}}\}\right)}^{2}+2ℑ\left\{h_{\left(d\right),tl_{2}}\right\}\sum_{p_{3}=1}^{N}\sum_{q_{3}=1}^{N}\left|h_{l_{2}p_{3}}\right|\left|g_{q_{3}t}\right|\sin\delta_{p_{3}q_{3}}\right)\right], \end{array}$$

by expanding this equation and removing the null terms, then $E\left[s_{l_{1}i}^{2}s_{l_{2}t}^{2}\right]$ can be obtained by (A79).

(A79) $$\begin{array}{c} E\left[s_{l_{1}i}^{2}s_{l_{2}t}^{2}\right]=\sum_{p_{1}=1}^{N}\sum_{p_{2}=1}^{N}\sum_{p_{3}=1}^{N}\sum_{p_{4}=1}^{N}\sum_{q_{1}=1}^{N}\sum_{q_{2}=1}^{N}\sum_{q_{3}=1}^{N}\sum_{q_{4}=1}^{N}E\left[\left|h_{l_{1}p_{1}}\right|\left|h_{l_{1}p_{2}}\right|\left|h_{l_{2}p_{3}}\right|\left|h_{l_{2}p_{4}}\right|\right]\times \\ E\left[\left|g_{q_{1}i}\right|\left|g_{q_{2}i}\right|\right]E\left[\left|g_{q_{3}t}\right|\left|g_{q_{4}t}\right|\right]E\left[\sin\delta_{p_{1}q_{1}}\sin\delta_{p_{2}q_{2}}\sin\delta_{p_{3}q_{3}}\sin\delta_{p_{4}q_{4}}\right]+\\ \xi_{ℑ\{h_{\left(d\right)}\}}\sum_{p_{1}=1}^{N}\sum_{p_{2}=1}^{N}\sum_{q_{1}=1}^{N}\sum_{q_{2}=1}^{N}E\left[\left|h_{l_{1}p_{1}}\right|\left|h_{l_{1}p_{2}}\right|\right]E\left[\left|g_{q_{1}i}\right|\left|g_{q_{2}i}\right|\right]E\left[\sin\delta_{p_{1}q_{1}}\sin\delta_{p_{2}q_{2}}\right]+\\ \xi_{ℑ\{h_{\left(d\right)}\}}\sum_{p_{3}=1}^{N}\sum_{p_{4}=1}^{N}\sum_{q_{3}=1}^{N}\sum_{q_{4}=1}^{N}E\left[\left|h_{l_{2}p_{3}}\right|\left|h_{l_{2}p_{4}}\right|\right]E\left[\left|g_{q_{3}t}\right|\left|g_{q_{4}t}\right|\right]E\left[\sin\delta_{p_{3}q_{3}}\sin\delta_{p_{4}q_{4}}\right]+\\ E\left[{\left(ℑ\{h_{\left(d\right),il_{1}}\}\right)}^{2}\right]E\left[{\left(ℑ\{h_{\left(d\right),tl_{2}}\}\right)}^{2}\right] \end{array}$$

by expanding the terms, it is easy to note that many expressions are equal to zero because of the expected value of the sine of a zero mean Von Mises random variable. The fourth-order trigonometric moment of the phase is $E\left[\sin^{4}\delta \right]=1-2b_{1}+b_{2}$, since $\sin^{4}\delta ={\left(1-\cos^{2}\delta \right)}^{2}=1-2\cos\delta \cos^{4}\delta$, therefore

(A80) $$E\left[s_{l_{1}i}^{2}s_{l_{2}t}^{2}\right]=N^{2}\xi_{h}^{2}\xi_{g}^{2}\left(1-2b_{1}+b_{2}\right)+2N^{2}\xi_{ℑ\{h_{\left(d\right)}\}}\xi_{h}\xi_{g}b_{29}+\xi_{ℑ\{h_{\left(d\right)}\}}^{2},\forall \;l_{1}\neq l_{2},$$

(A81) $$E\left[s_{l_{1}i}^{2}s_{l_{2}t}^{2}\right]=N^{2}\epsilon_{h}\epsilon_{g}\left(1-2b_{1}+b_{2}\right)+2N^{2}\xi_{ℑ\{h_{\left(d\right)}\}}\xi_{h}\xi_{g}b_{29}+\xi_{ℑ\{h_{\left(d\right)}\}}^{2},\forall \;l_{1}=l_{2},$$

(A82) $$\begin{array}{c} \mu_{S_{i}S_{t}}=E\left[SiSt\right]=M\left(M-1\right)\left[N^{2}\xi_{h}^{2}\xi_{g}^{2}\left(1-2b_{1}+b_{2}\right)+2N^{2}\xi_{ℑ\{h_{\left(d\right)}\}}\xi_{h}\xi_{g}b_{29}+\xi_{ℑ\{h_{\left(d\right)}\}}^{2}\right]+\\ \;M\left[N^{2}\epsilon_{h}\epsilon_{g}\left(1-2b_{1}+b_{2}\right)+2N^{2}\xi_{ℑ\{h_{\left(d\right)}\}}\xi_{h}\xi_{g}b_{29}\right]. \end{array}$$

The expected value $E\left[S_{i}C_{t}\right]$, considering that i≠t, can be computed as

(A83) $$E\left[S_{i}C_{t}\right]=E\left[\sum_{l_{1}=1}^{M}s_{l_{1}i}^{2}\sum_{l_{2}=1}^{M}c_{l_{2}t}^{2}\right]=\sum_{l_{1}=1}^{M}\sum_{l_{2}=1}^{M}E\left[s_{l_{1}i}^{2}c_{l_{2}t}^{2}\right],$$

where $E\left[s_{l_{1}i}^{2}c_{l_{2}t}^{2}\right]$ can be defined as

(A84) $$\begin{array}{c} E\left[s_{l_{1}i}^{2}c_{l_{2}t}^{2}\right]=E\left[\left(\sum_{p_{1}=1}^{N}\sum_{p_{2}=1}^{N}\sum_{q_{1}=1}^{N}\sum_{q_{2}=1}^{N}\left|h_{l_{1}p_{1}}\right|\left|h_{l_{1}p_{2}}\right|\left|g_{q_{1}i}\right|\left|g_{q_{2}i}\right|\sin\delta_{p_{1}q_{1}}\sin\delta_{p_{2}q_{2}}+\right.\right.\\ \left.\left.{\left(ℑ\{h_{\left(d\right),il_{1}}\}\right)}^{2}+2ℑ\left\{h_{\left(d\right),il_{1}}\right\}\sum_{p_{1}=1}^{N}\sum_{q_{1}=1}^{N}\left|h_{l_{1}p_{1}}\right|\left|g_{q_{1}i}\right|\sin\delta_{p_{1}q_{1}}\right)\times \right.\\ \left.\left(\sum_{p_{3}=1}^{N}\sum_{p_{4}=1}^{N}\sum_{q_{3}=1}^{N}\sum_{q_{4}=1}^{N}\left|h_{l_{2}p_{3}}\right|\left|h_{l_{2}p_{4}}\right|\left|g_{q_{3}t}\right|\left|g_{q_{4}t}\right|\cos\delta_{p_{3}q_{3}}\cos\delta_{p_{4}q_{4}}+{\left(ℜ\{h_{\left(d\right),tl_{2}}\}\right)}^{2}+\right.\right.\\ \left.\left.2ℜ\left\{h_{\left(d\right),tl_{2}}\right\}\sum_{p_{3}=1}^{N}\sum_{q_{3}=1}^{N}\left|h_{l_{2}p_{3}}\right|\left|g_{q_{3}t}\right|\cos\delta_{p_{3}q_{3}}\right)\right], \end{array}$$

by expanding and removing the null terms, then $E\left[s_{l_{1}i}^{2}c_{l_{2}t}^{2}\right]$ can be calculated by (A85).

(A85) $$\begin{array}{c} E\left[s_{l_{1}i}^{2}c_{l_{2}t}^{2}\right]=\sum_{p_{1}=1}^{N}\sum_{p_{3}=1}^{N}\sum_{p_{4}=1}^{N}\sum_{q_{1}=1}^{N}\sum_{q_{2}=1}^{N}\sum_{q_{3}=1}^{N}\sum_{q_{4}=1}^{N}E\left[\left|h_{l_{1}p_{1}}\right|\left|h_{l_{1}p_{1}}\right|\left|h_{l_{2}p_{3}}\right|\left|h_{l_{2}p_{4}}\right|\right]\times \\ E\left[\left|g_{q_{1}i}\right|\left|g_{q_{2}i}\right|\left|g_{q_{3}t}\right|\left|g_{q_{4}t}\right|\right]E\left[\cos\delta_{p_{3}q_{3}}\cos\delta_{p_{4}q_{4}}\right]E\left[\sin\delta_{p_{1}q_{1}}\sin\delta_{p_{1}q_{2}}\right]+\\ \xi_{ℜ\{h_{\left(d\right)}\}}\sum_{p_{1}=1}^{N}\sum_{q_{1}=1}^{N}\sum_{q_{2}=1}^{N}E\left[\left|h_{l_{1}p_{1}}\right|\left|h_{l_{1}p_{1}}\right|\right]E\left[\left|g_{q_{1}i}\right|\left|g_{q_{2}i}\right|\right]E\left[\sin\delta_{p_{1}q_{1}}\sin\delta_{p_{1}q_{2}}\right]+\\ \xi_{ℑ\{h_{\left(d\right)}\}}\sum_{p_{3}=1}^{N}\sum_{q_{3}=1}^{N}\sum_{q_{4}=1}^{N}E\left[\left|h_{l_{2}p_{3}}\right|\left|h_{l_{2}p_{3}}\right|\right]E\left[\left|g_{q_{3}t}\right|\left|g_{q_{4}t}\right|\right]E\left[\cos\delta_{p_{3}q_{3}}\cos\delta_{p_{3}q_{4}}\right]+\xi_{ℜ\{h_{\left(d\right)}\}}+\\ \left(N-1\right)\xi_{ℑ\{h_{\left(d\right)}\}}\sum_{p_{3}=1}^{N}\sum_{q_{3}=1}^{N}\sum_{q_{4}=1}^{N}\mu_{h}^{2}E\left[\left|g_{q_{3}t}\right|\left|g_{q_{4}t}\right|\right]\alpha_{1}^{2}+\xi_{ℜ\{h_{\left(d\right)}\}} \end{array}$$

by expanding the summations and evaluating the expected values recursively, it follows that, for i≠t,

(A86) $$\begin{array}{c} E\left[s_{l_{1}i}^{2}c_{l_{2}t}^{2}\right]=b_{12}b_{27}\mu_{g}^{2}\xi_{g}b_{28}+N^{2}b_{14}b_{17}b_{29}\alpha_{1}^{2}+N^{2}b_{14}b_{27}\mu_{g}^{2}\xi_{g}b_{28}+Nb_{14}b_{27}\mu_{g}^{2}\xi_{g}b_{28}+\\ b_{12}b_{27}\xi_{g}^{2}b_{28}+{\left(N-1\right)}^{4}b_{10}b_{18}+b_{12}b_{27}\xi_{g}^{2}b_{28}+b_{12}b_{27}b_{23}b_{28}+4b_{8}b_{17}b_{28}+N^{2}\left(N-1\right)\xi_{g}^{2}b_{27}b_{28}+\\ b_{12}b_{17}b_{28}+N^{2}\left(N-1\right)\xi_{g}^{2}b_{27}b_{28}+b_{8}b_{23}\xi_{h}^{2}b_{28}\left(b_{8}+N^{2}\left(N-1\right)\right)+N^{2}\left(N-1\right)\mu_{g}^{2}\xi_{g}\xi_{h}^{2}b_{28}+\\ N^{2}\xi_{h}^{2}\xi_{g}^{2}b_{28}+\left(N-1\right)N^{2}\xi_{ℜ\{h_{\left(d\right)}\}}\xi_{h}\mu_{g}^{2}b_{29}+N^{2}\xi_{ℜ\{h_{\left(d\right)}\}}\xi_{h}^{2}b_{29}+\xi_{ℑ\{h_{\left(d\right)}\}}N^{2}(\left(N-1\right)b_{25}\alpha_{1}^{2}+\\ \xi_{h}\xi_{g}b_{1})+\xi_{ℜ\{h_{\left(d\right)}\}}+b_{8}\xi_{ℑ\{h_{\left(d\right)}\}}b_{24}+N^{2}\left(N-1\right)\xi_{ℑ\{h_{\left(d\right)}\}}\mu_{h}^{2}\xi_{g}\alpha_{1}^{2}+2\xi_{ℜ\{h_{\left(d\right)}\}},\forall l_{1}\neq l_{2}, \end{array}$$

and $E\left[s_{l_{1}i}^{2}c_{l_{1}t}^{2}\right]$ can be obtained by (A87).

(A87) $$\begin{array}{c} E\left[s_{l_{1}i}^{2}c_{l_{1}t}^{2}\right]={\left(N-1\right)}^{4}b_{10}b_{18}+N^{2}b_{14}b_{17}b_{29}\alpha_{1}^{2}+Nb_{14}b_{27}\mu_{g}^{2}\xi_{g}b_{28}+N^{2}b_{14}b_{27}\mu_{g}^{2}\xi_{g}b_{28}+\\ b_{12}b_{27}\xi_{g}^{2}b_{28}+b_{12}b_{27}\mu_{g}^{2}\xi_{g}b_{28}+b_{12}b_{27}\xi_{g}^{2}b_{28}+b_{12}\mu_{h}^{2}\xi_{h}b_{23}b_{28}+b_{8}\mu_{h}^{2}\xi_{h}\mu_{g}^{2}\xi_{g}b_{28}+b_{8}\chi_{h}\mu_{h}\xi_{g}\mu_{g}^{2}b_{28}+\\ N^{2}\left(N-1\right)\mu_{h}\chi_{h}\xi_{g}^{2}b_{28}+b_{12}\mu_{g}^{2}\chi_{h}\mu_{h}\xi_{g}b_{28}+2b_{8}\mu_{g}^{2}\xi_{g}\chi_{h}\mu_{h}b_{28}+N^{2}\left(N-1\right)\chi_{h}\mu_{h}\xi_{g}^{2}b_{28}+b_{8}b_{23}\epsilon_{h}b_{28}+\\ N^{2}\left(N-1\right)b_{23}\epsilon_{h}b_{28}+N^{2}\left(N-1\right)\mu_{g}^{2}\xi_{g}\epsilon_{h}b_{28}+N^{2}E\chi_{h}\xi_{g}^{2}b_{28}+\left(N-1\right)N^{2}\xi_{ℜ\{h_{\left(d\right)}\}}\xi_{h}\mu_{g}^{2}b_{29}+\\ \xi_{ℜ\{h_{\left(d\right)}\}}N^{2}\xi_{h}\xi_{g}b_{29}+\xi_{ℑ\{h_{\left(d\right)}\}}N^{2}\left(\left(N-1\right)b_{25}\alpha_{1}^{2}+\xi_{h}\xi_{g}b_{1}\right)+\xi_{ℜ\{h_{\left(d\right)}\}}+N^{2}\left(N-1\right)\xi_{ℑ\{h_{\left(d\right)}\}}\mu_{h}^{2}\xi_{g}\alpha_{1}^{2}+\\ b_{8}\xi_{ℑ\{h_{\left(d\right)}\}}b_{24}+2\xi_{ℜ\{h_{\left(d\right)}\}},\forall i\neq t,l_{1}=l_{2}. \end{array}$$

Therefore, the term $E\left[S_{i}C_{t}\right]$ is given by (A88).

(A88) $$\begin{array}{c} \mu_{S_{i}C_{t}}=E\left[S_{i}C_{t}\right]=M\left(M-1\right)\left[{\left(N-1\right)}^{4}b_{10}b_{18}+N^{2}b_{14}b_{17}b_{29}\alpha_{1}^{2}+Nb_{14}b_{27}\mu_{g}^{2}\xi_{g}b_{28}+2\xi_{ℜ\{h_{\left(d\right)}\}}+\right.\\ \left.N^{2}b_{14}b_{27}\mu_{g}^{2}\xi_{g}b_{28}+b_{12}b_{27}\xi_{g}^{2}b_{28}+b_{12}b_{27}\mu_{g}^{2}\xi_{g}b_{28}+b_{12}b_{27}\xi_{g}^{2}b_{28}+b_{12}b_{27}b_{23}b_{28}+4b_{8}b_{17}b_{28}+\right.\\ \left.N^{2}\left(N-1\right)\xi_{g}^{2}b_{27}b_{28}+b_{12}b_{17}b_{28}+N^{2}\left(N-1\right)\xi_{g}^{2}b_{27}b_{28}+b_{8}b_{23}\xi_{h}^{2}b_{28}\left(b_{8}+N^{2}\left(N-1\right)\right)+\right.\\ \left.N^{2}\left(N-1\right)\mu_{g}^{2}\xi_{g}\xi_{h}^{2}b_{28}+N^{2}\xi_{h}^{2}\xi_{g}^{2}b_{28}+\left(N-1\right)N^{2}\xi_{ℜ\{h_{\left(d\right)}\}}\xi_{h}\mu_{g}^{2}b_{29}+\xi_{ℜ\{h_{\left(d\right)}\}}N^{2}\xi_{h}^{2}b_{29}+\right.\\ \left.\xi_{ℑ\{h_{\left(d\right)}\}}N^{2}\left(\left(N-1\right)b_{25}\alpha_{1}^{2}+\xi_{h}\xi_{g}b_{1}\right)+\xi_{ℜ\{h_{\left(d\right)}\}}+b_{8}\xi_{ℑ\{h_{\left(d\right)}\}}b_{24}+N^{2}\left(N-1\right)\xi_{ℑ\{h_{\left(d\right)}\}}\mu_{h}^{2}\xi_{g}\alpha_{1}^{2}\right]+\\ M\left[{\left(N-1\right)}^{4}b_{10}b_{18}+N^{2}b_{14}b_{17}b_{29}\alpha_{1}^{2}+Nb_{14}b_{27}\mu_{g}^{2}\xi_{g}b_{28}+N^{2}b_{14}b_{27}\mu_{g}^{2}\xi_{g}b_{28}+b_{12}b_{27}\xi_{g}^{2}b_{28}+\right.\\ \left.b_{12}b_{27}b_{28}\left(\mu_{g}^{2}\xi_{g}+\xi_{g}^{2}\right)+b_{12}\mu_{h}^{2}\xi_{h}b_{23}b_{28}+b_{8}\mu_{h}^{2}\xi_{h}\mu_{g}^{2}\xi_{g}b_{28}+b_{8}\chi_{h}\mu_{h}\xi_{g}\mu_{g}^{2}b_{28}+N^{2}E\chi_{h}\xi_{g}^{2}b_{28}+\right.\\ \left.N^{2}\left(N-1\right)\mu_{h}\chi_{h}\xi_{g}^{2}b_{28}+b_{12}\mu_{g}^{2}\chi_{h}\mu_{h}\xi_{g}b_{28}+2b_{8}\mu_{g}^{2}\xi_{g}\chi_{h}\mu_{h}b_{28}+\xi_{ℜ\{h_{\left(d\right)}\}}b_{8}\xi_{ℑ\{h_{\left(d\right)}\}}b_{24}+\right.\\ \left.N^{2}\left(N-1\right)\chi_{h}\mu_{h}\xi_{g}^{2}b_{28}+b_{8}b_{23}\epsilon_{h}b_{28}+N^{2}\left(N-1\right)b_{23}\epsilon_{h}b_{28}+N^{2}\left(N-1\right)\mu_{g}^{2}\xi_{g}\epsilon_{h}b_{28}+2\xi_{ℜ\{h_{\left(d\right)}\}}+\right.\\ \left.\left(N-1\right)N^{2}\xi_{ℜ\{h_{\left(d\right)}\}}\xi_{h}\mu_{g}^{2}b_{29}+\xi_{ℜ\{h_{\left(d\right)}\}}N^{2}\xi_{h}\xi_{g}b_{29}+\xi_{ℑ\{h_{\left(d\right)}\}}N^{2}\left(\left(N-1\right)b_{25}\alpha_{1}^{2}+\xi_{h}\xi_{g}b_{1}\right)+\right.\\ \left.N^{2}\left(N-1\right)\xi_{ℑ\{h_{\left(d\right)}\}}\mu_{h}^{2}\xi_{g}\alpha_{1}^{2}\right]. \end{array}$$

Since

(A89) $$E\left[Z_{i}Z_{t}\right]={\left(\frac{P}{K}\right)}^{2}\left(\mu_{C_{i}C_{t}}+\mu_{S_{i}S_{t}}+2\mu_{S_{i}C_{t}}\right),$$

it follows that

(A90) $$cov\left(Z_{i},Z_{t}\right)={\left(\frac{P}{K}\right)}^{2}\left(\mu_{C_{i}C_{t}}+\mu_{S_{i}S_{t}}+2\mu_{S_{i}C_{t}}\right)-\mu_{Z_{k}}^{2}.$$

Therefore,

(A91) $$\sigma_{F_{k}}^{2}=K\left(K-1\right)\left({\left(\frac{P}{K}\right)}^{2}\left(\mu_{C_{i}C_{t}}+\mu_{S_{i}S_{t}}+2\mu_{S_{i}C_{t}}\right)-\mu_{Z_{k}}^{2}\right)+K\sigma_{Z_{k}}^{2}.$$

## Appendix C. Statistical Parameters of *γ_k_*

### Appendix C.1. Expected Value of γ_k_

The expected value of $\gamma_{k}$, considering the gamma distribution in

(A92) $$\mu_{\gamma_{k}}=E\left[\gamma_{k}\right]=\frac{\mu_{Z_{k}}}{\mu_{F_{k}}},$$

since $\mu_{Z_{k}}$ and $\mu_{F_{k}}$ are already known, can be computed using the derived expressions.

### Appendix C.2. Variance of γ_k_

The variance of $\gamma_{k}$ can be computed considering the moments of $F_{k}$ and $Z_{k}$ as

(A93) $$\sigma_{\gamma_{k}}^{2}={\left(\frac{\mu_{Z_{k}}}{\mu_{F_{k}}}\right)}^{2}\left[\frac{\sigma_{Z_{k}}^{2}}{\mu_{Z_{k}}^{2}}-2\left(\frac{\left(K-1\right)cov\left(Z_{i},Z_{k}\right)}{\mu_{Z_{k}}\mu_{F_{k}}}\right)+\frac{\sigma_{F_{k}}^{2}}{\mu_{F_{k}}^{2}}\right],$$

since $\mu_{Z_{k}}$, $\mu_{F_{k}}$, $\sigma_{Z_{k}}^{2}$, $\sigma_{F_{k}}^{2}$ and $cov(Z_{i},Z_{k})$ were previously derived, these parameters can be substituted here.
